# Supplementary material for: Proton export upregulates aerobic glycolysis
Source: BMC Biol. 2022 Jul 15;20:163. doi: 10.1186/s12915-022-01340-0 (PMC9287933; doi:10.1186/s12915-022-01340-0)
Supplement: Supplementary file 1 — Additional file 1: Table S1. Average intracellular pHi in control and CA-IX expressing MCF-7 cells as a function of extracellular pH. n=158-438 cells , standard deviation shown. Table S2. Effect of PMA1 expression on experimental and spontaneous metastasis in MCF-7 cells. (Two-tailed Fisher’s exact t-test p<0.05*). Figure S1. Uptake of 3H-2-deoxy glucose in control and CAIX MCF-7 clones (N=3; Ordinary one-way ANOVA *, ***, **** p<0.05, 0.005, 0.001). Figure S2. YSI analysis of lactate in media at 24 and 48 hours in CA-IX clones (N=6 replicates, Ordinary one-way ANOVA p<0.01**, 0.005***, 0.001****.). Figure S3. ATP linked OCR measured by mitochondrial stress test XFe96 Seahorse assay, by injecting oligomycin to shut off mitochondrial respiration. (N=8 replicates, ordinary one-way ANOVA p<0.001****.). Figure S4. Mitochondrial polarization using JC-1 mitochondrial dye in CA-IX clones (N=8-12 per group, 2 bioreplicates; Kruskal Wallis Test p<0.05*, p<0.01**, p<0.005***). Figure S5. Lactate production measured by YSI and Western blot of CA-IX transfected U2-OS cells after 2hrs. (N=9; Ordinary one way ANOVA, p<0.05*, p<0.01**, p<0.005***, p<0.001****.). Figure S6. Lactate production measured by YSI and Western blot of CA-IX transfected HEK 293T cells after 1hr. (N=9; Ordinary one way ANOVA, p<0.05*, p<0.01**, p<0.005***, p<0.001****.). Figure S7. In vitro growth rates of MCF-7, MOCK-2 and CA-IX clones M1 and M6. n= 3, average ± SD. p<0.0001****. Figure S8. Effect of chloride vs gluconate on glucose induced PPR at different pHe. Average glucose induced PPR ± SD, n=8, unpaired t-test p<0.005***, p<0.0001****. Figure S9. cSNARF1 calibration curve with nigericin/high K+. Figure S10. Migration assay to measure effects of CA-IX expression on migration of MCF7 cells. Imaged using Celigo, n=3 bio-replicates in triplicate, average ± SD. (Ordinary one-way ANOVA, p<0.05*, p<0.005***,p<0.0001****). Figure S11. Gel escape assay to measure effects of CA-IX expression on invasion and mi [file 12915_2022_1340_MOESM1_ESM.pptx]

## Slide 1
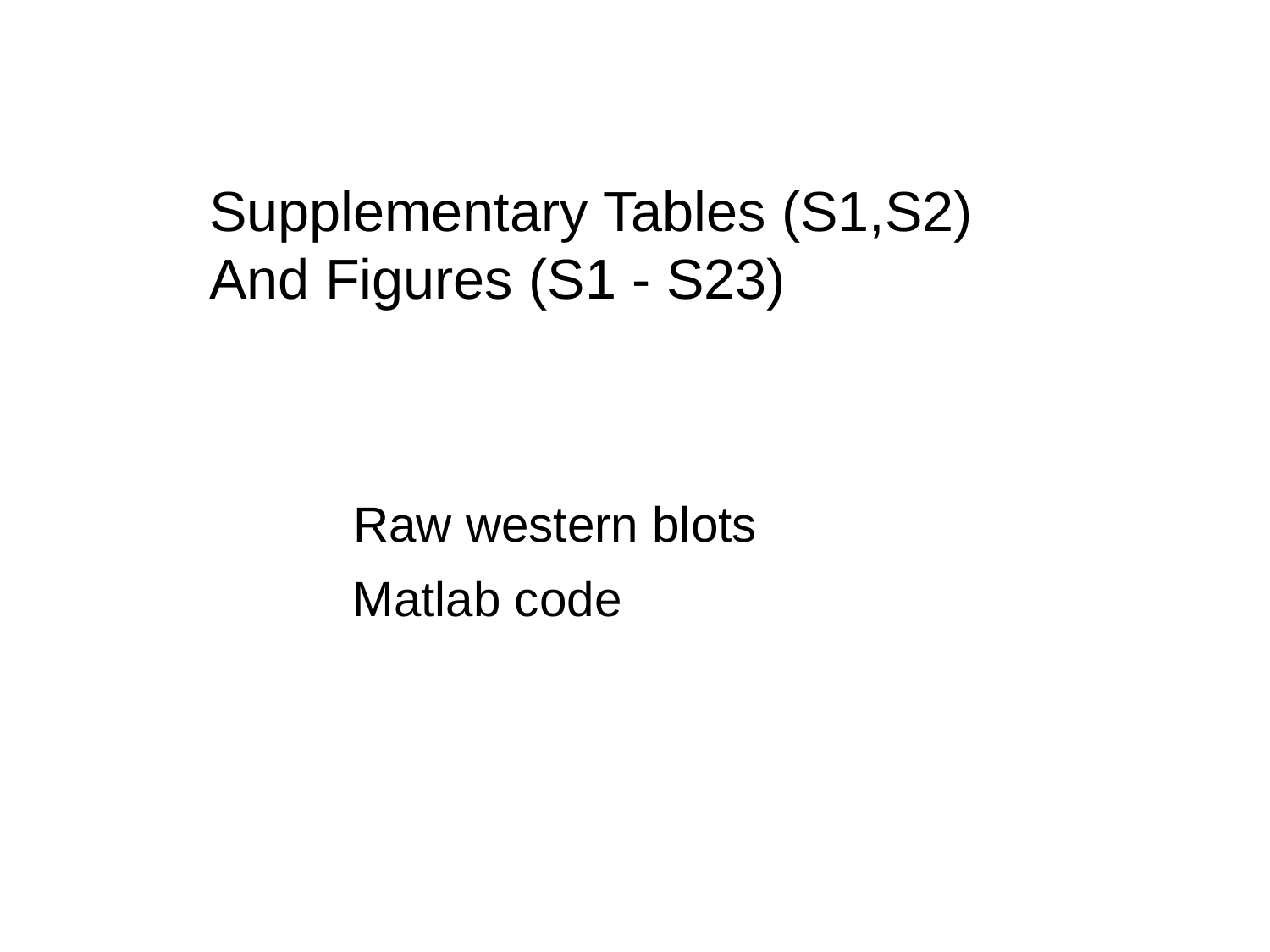

Supplementary Tables (S1,S2)
And Figures (S1 - S23)
Raw western blots
Matlab code

## Slide 2
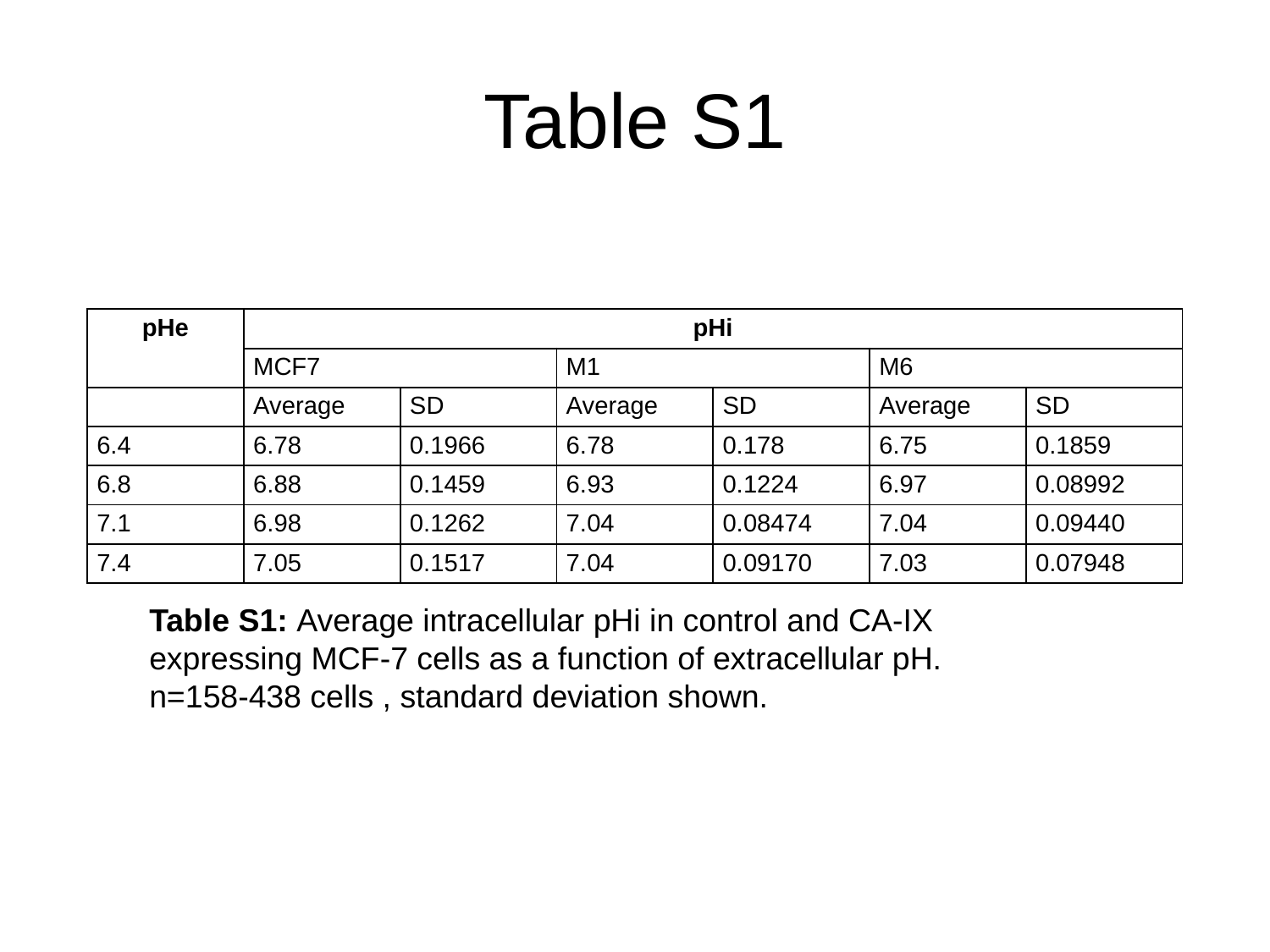

# Table S1
| pHe | pHi | | | | | |
| --- | --- | --- | --- | --- | --- | --- |
| | MCF7 | | M1 | | M6 | |
| | Average | SD | Average | SD | Average | SD |
| 6.4 | 6.78 | 0.1966 | 6.78 | 0.178 | 6.75 | 0.1859 |
| 6.8 | 6.88 | 0.1459 | 6.93 | 0.1224 | 6.97 | 0.08992 |
| 7.1 | 6.98 | 0.1262 | 7.04 | 0.08474 | 7.04 | 0.09440 |
| 7.4 | 7.05 | 0.1517 | 7.04 | 0.09170 | 7.03 | 0.07948 |
Table S1: Average intracellular pHi in control and CA-IX expressing MCF-7 cells as a function of extracellular pH. n=158-438 cells , standard deviation shown.

## Slide 3
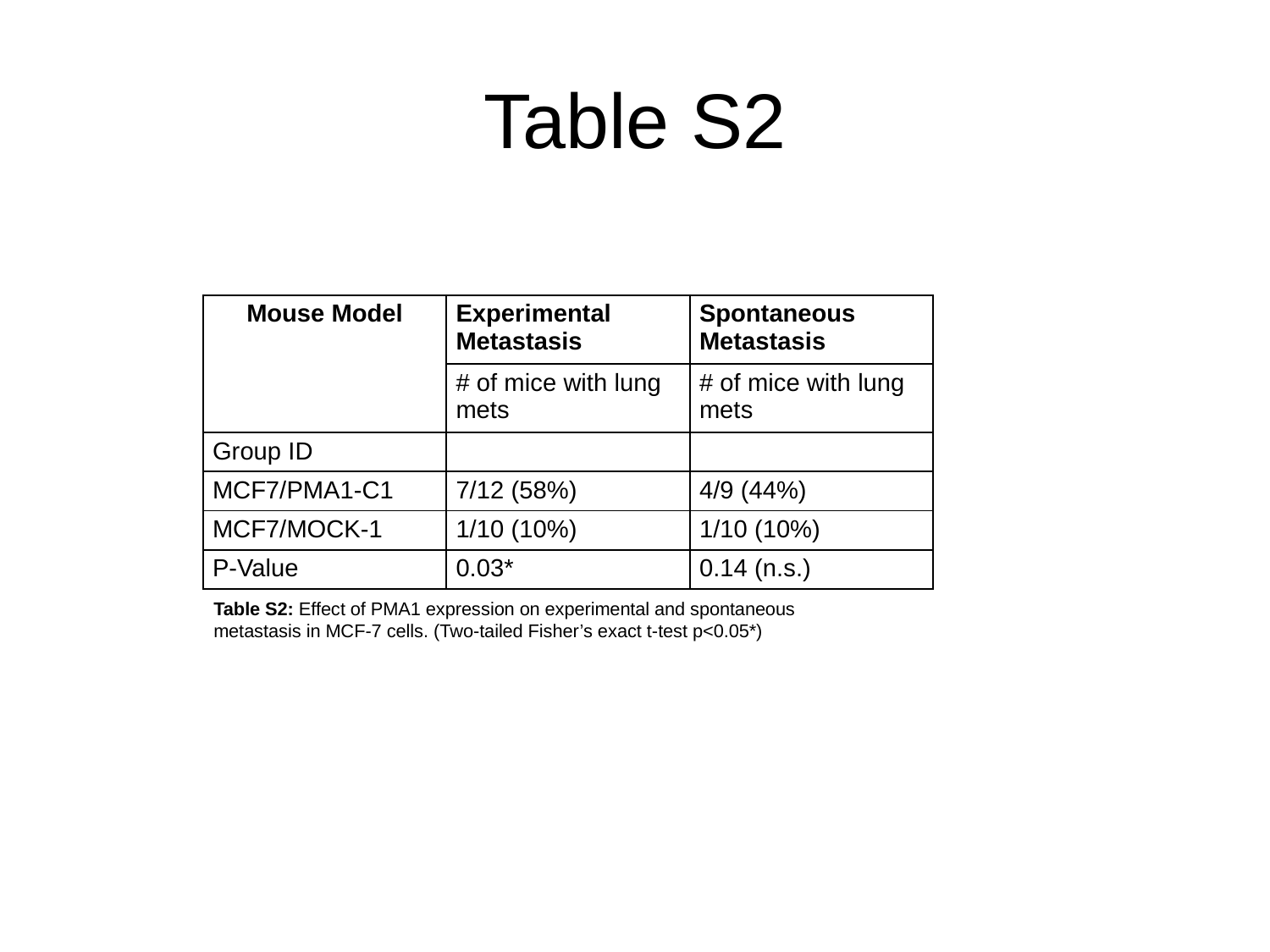

# Table S2
| Mouse Model | Experimental Metastasis | Spontaneous Metastasis |
| --- | --- | --- |
| | # of mice with lung mets | # of mice with lung mets |
| Group ID | | |
| MCF7/PMA1-C1 | 7/12 (58%) | 4/9 (44%) |
| MCF7/MOCK-1 | 1/10 (10%) | 1/10 (10%) |
| P-Value | 0.03\* | 0.14 (n.s.) |
Table S2: Effect of PMA1 expression on experimental and spontaneous metastasis in MCF-7 cells. (Two-tailed Fisher’s exact t-test p<0.05*)

## Slide 4
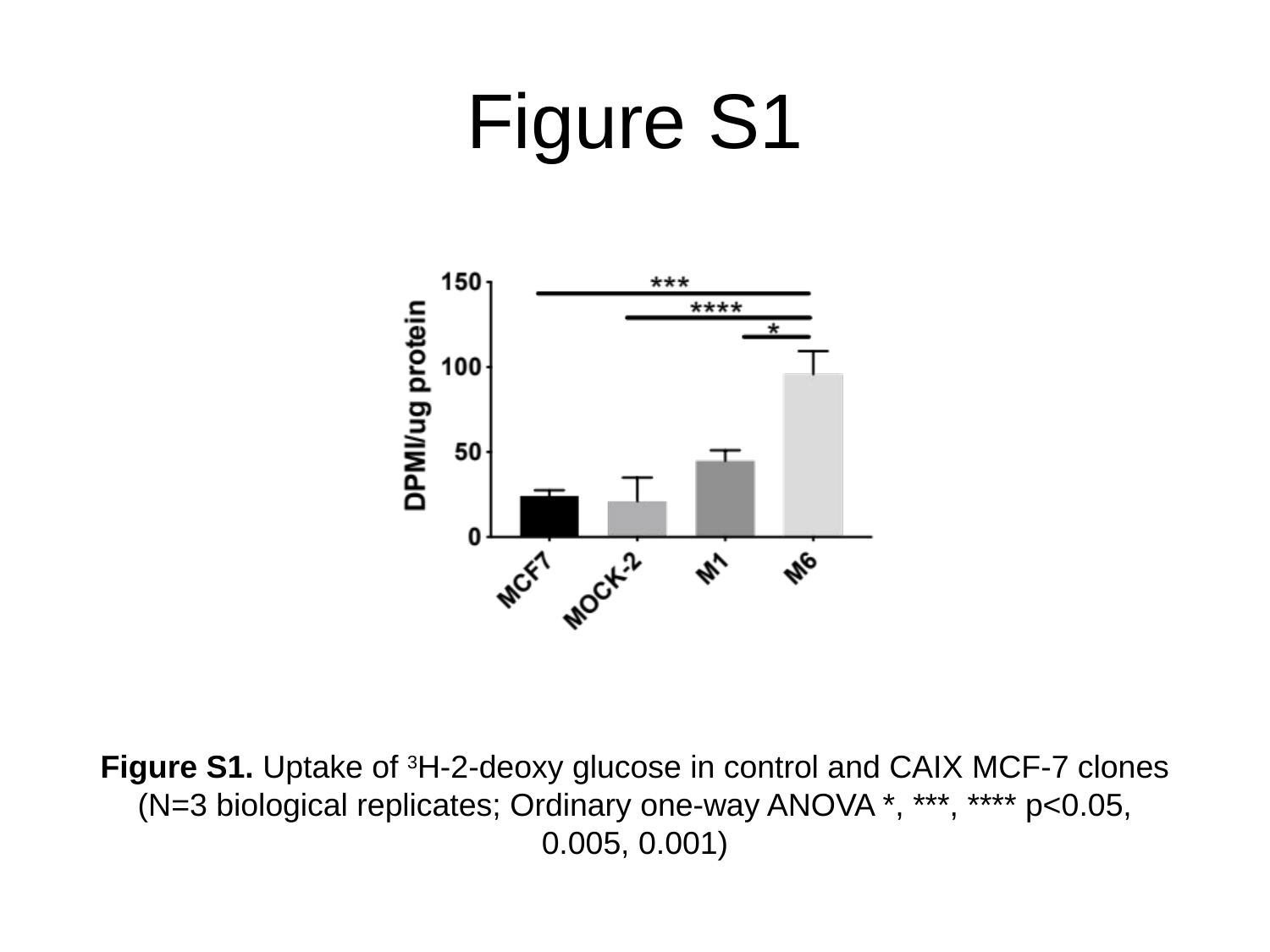

# Figure S1
Figure S1. Uptake of 3H-2-deoxy glucose in control and CAIX MCF-7 clones (N=3 biological replicates; Ordinary one-way ANOVA *, ***, **** p<0.05, 0.005, 0.001)

## Slide 5
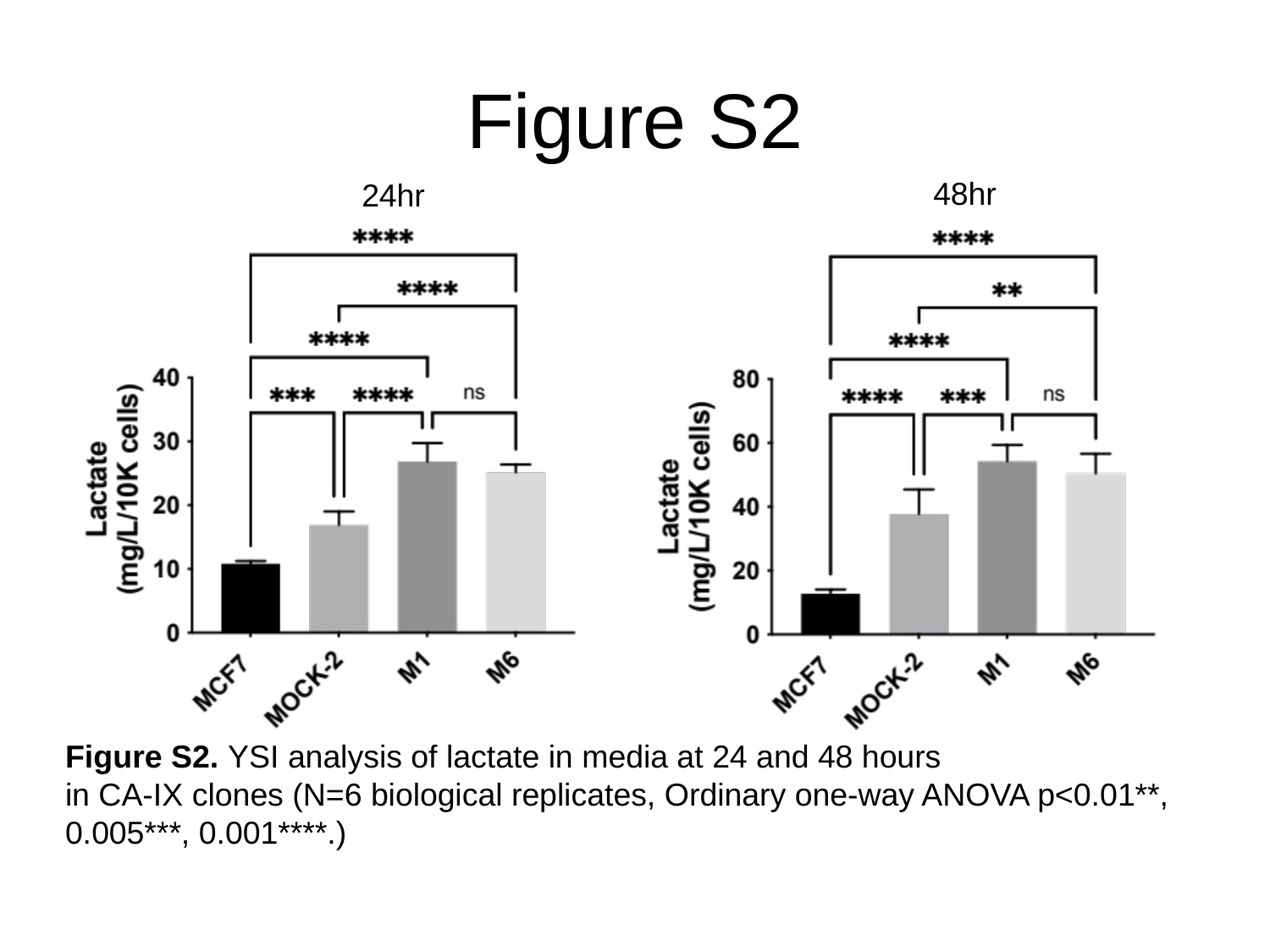

# Figure S2
48hr
24hr
Figure S2. YSI analysis of lactate in media at 24 and 48 hours
in CA-IX clones (N=6 biological replicates, Ordinary one-way ANOVA p<0.01**, 0.005***, 0.001****.)

## Slide 6
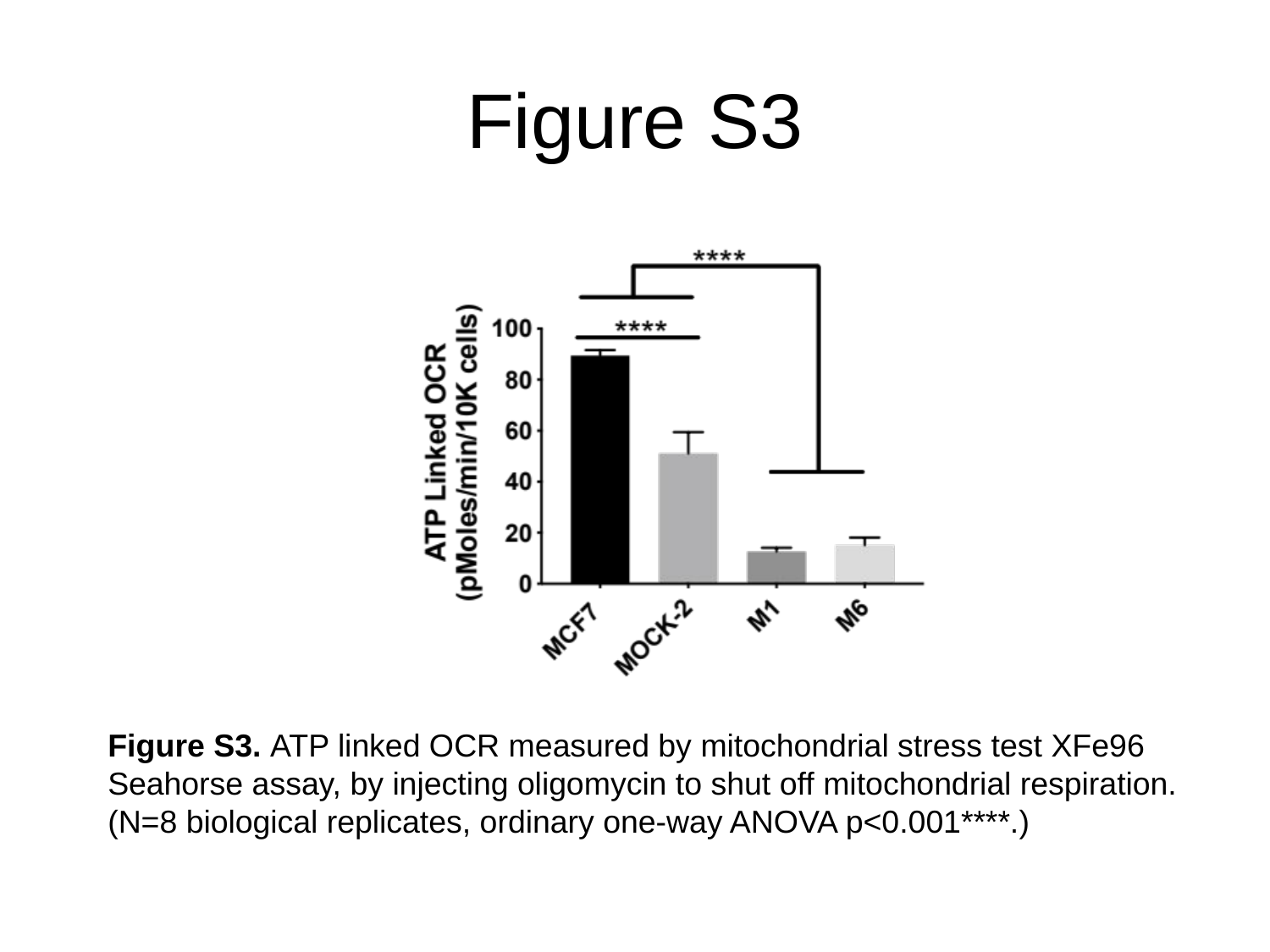

# Figure S3
Figure S3. ATP linked OCR measured by mitochondrial stress test XFe96 Seahorse assay, by injecting oligomycin to shut off mitochondrial respiration. (N=8 biological replicates, ordinary one-way ANOVA p<0.001****.)

## Slide 7
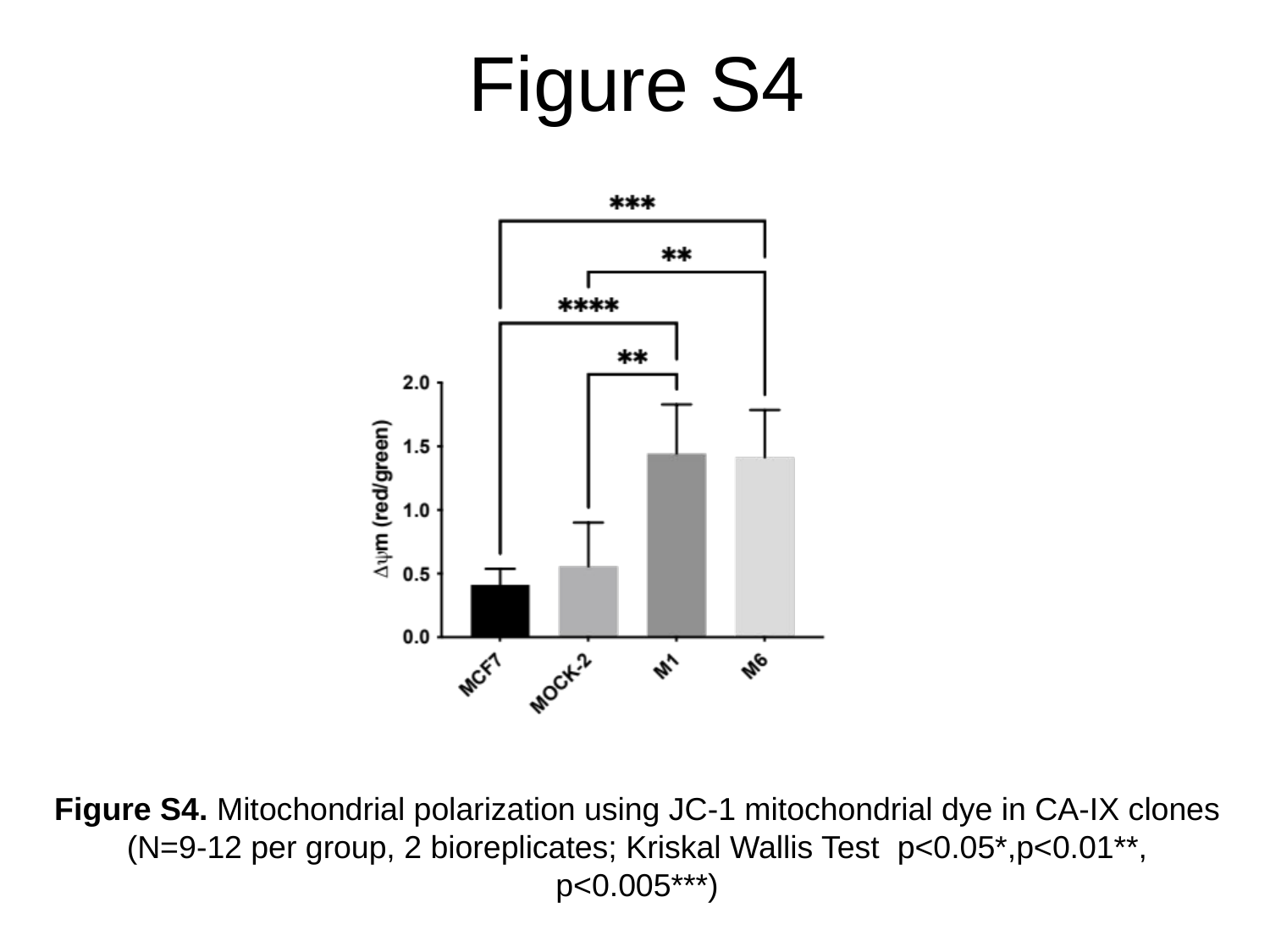

# Figure S4
Figure S4. Mitochondrial polarization using JC-1 mitochondrial dye in CA-IX clones (N=9-12 per group, 2 bioreplicates; Kriskal Wallis Test p<0.05*,p<0.01**, p<0.005***)

## Slide 8
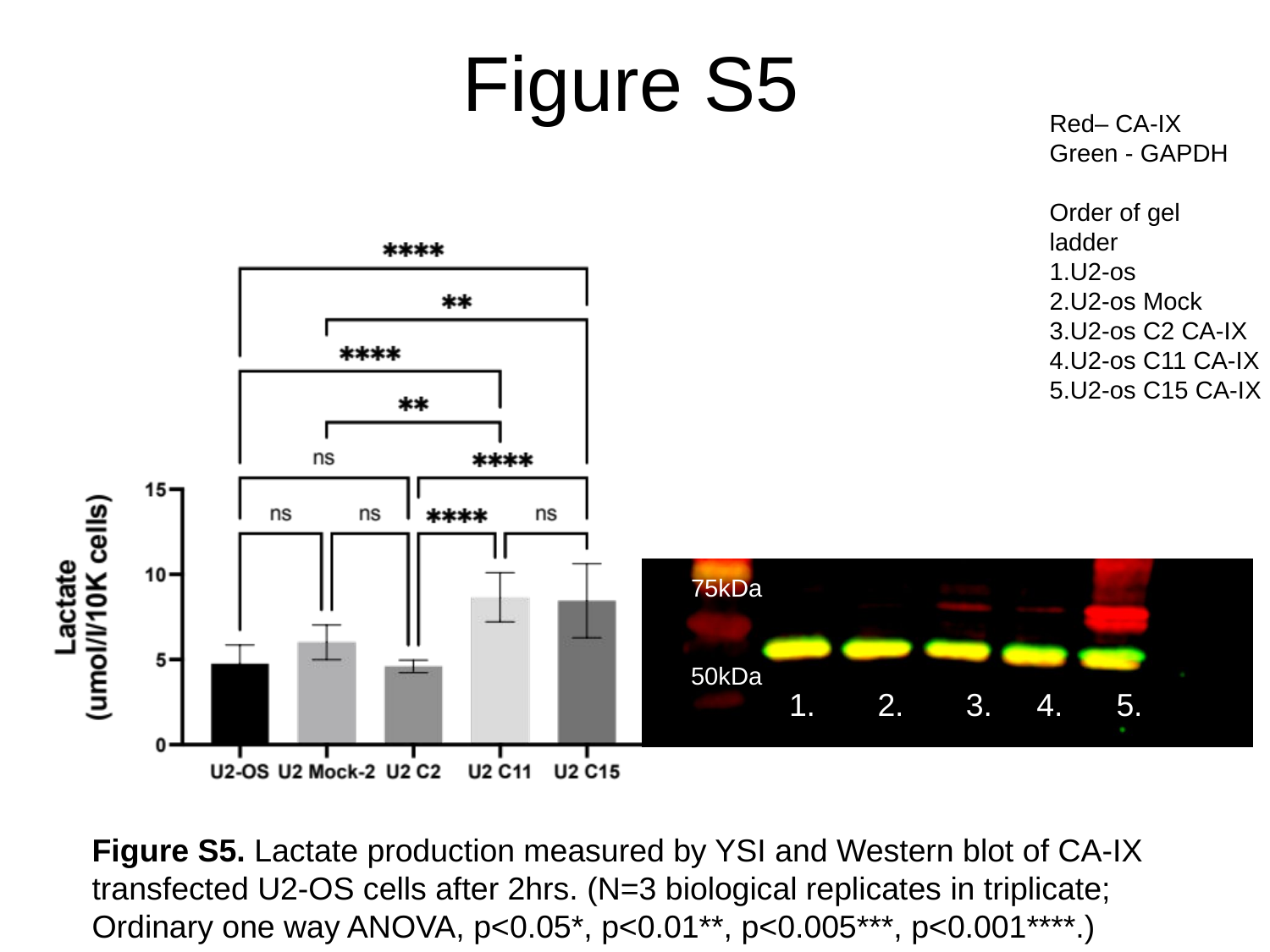

# Figure S5
Red– CA-IX
Green - GAPDH
Order of gel
ladder
1.U2-os
2.U2-os Mock
3.U2-os C2 CA-IX
4.U2-os C11 CA-IX
5.U2-os C15 CA-IX
75kDa
50kDa
1. 2. 3. 4. 5.
Figure S5. Lactate production measured by YSI and Western blot of CA-IX transfected U2-OS cells after 2hrs. (N=3 biological replicates in triplicate; Ordinary one way ANOVA, p<0.05*, p<0.01**, p<0.005***, p<0.001****.)

## Slide 9
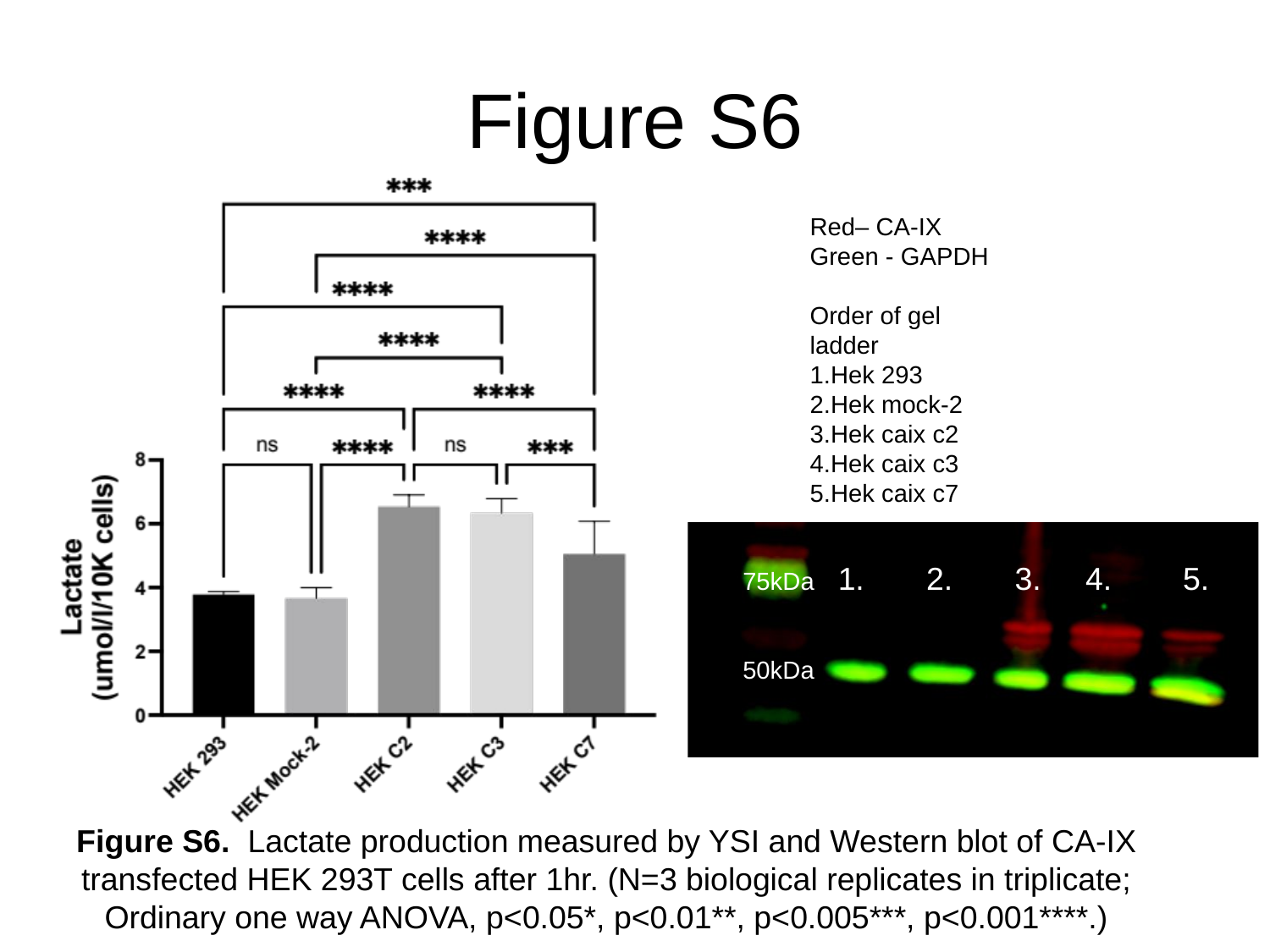

# Figure S6
Red– CA-IX
Green - GAPDH
Order of gel
ladder
1.Hek 293
2.Hek mock-2
3.Hek caix c2
4.Hek caix c3
5.Hek caix c7
1. 2. 3. 4. 5.
75kDa
50kDa
Figure S6. Lactate production measured by YSI and Western blot of CA-IX transfected HEK 293T cells after 1hr. (N=3 biological replicates in triplicate; Ordinary one way ANOVA, p<0.05*, p<0.01**, p<0.005***, p<0.001****.)

## Slide 10
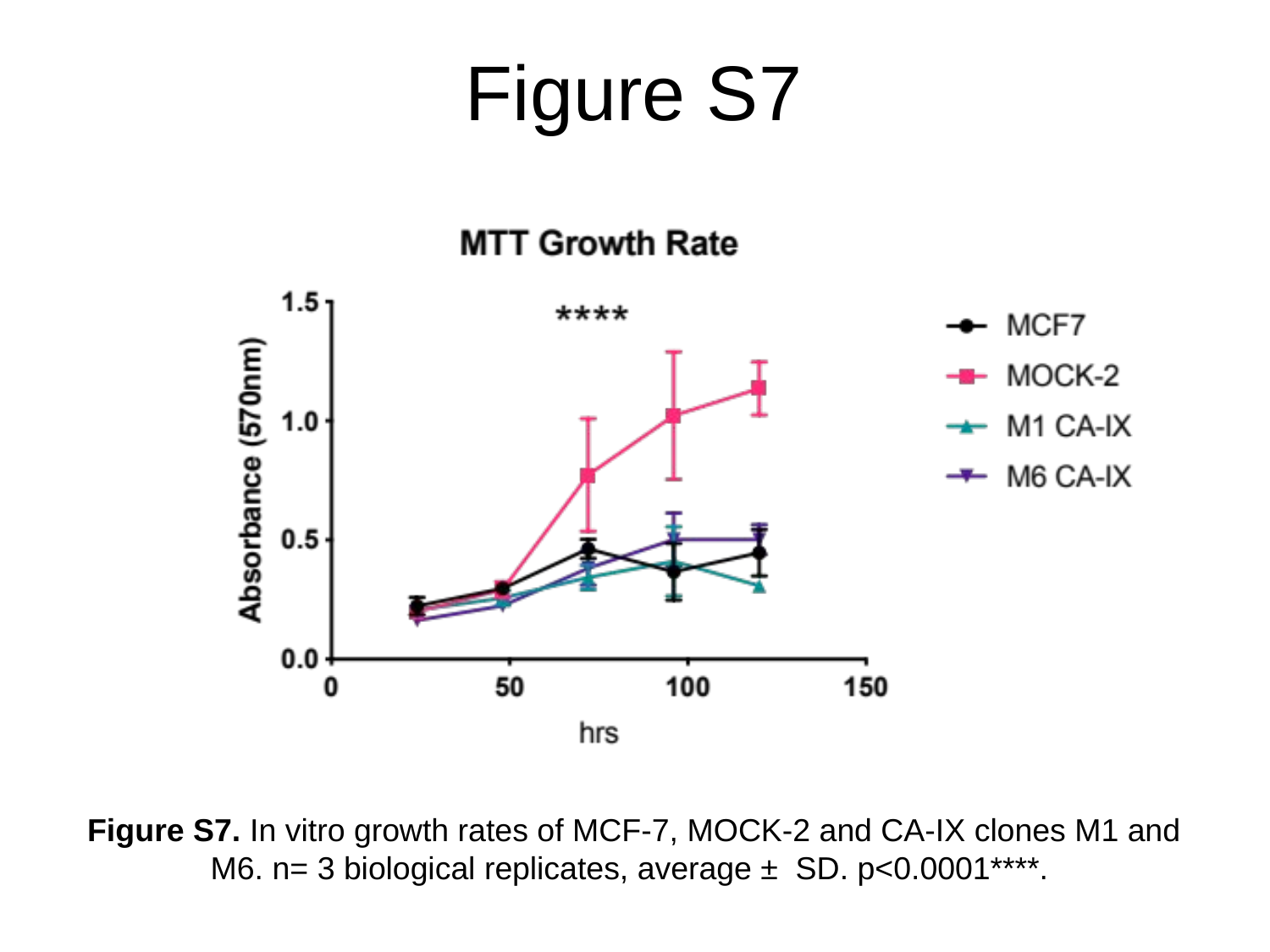

# Figure S7
Figure S7. In vitro growth rates of MCF-7, MOCK-2 and CA-IX clones M1 and M6. n= 3 biological replicates, average ± SD. p<0.0001****.

## Slide 11
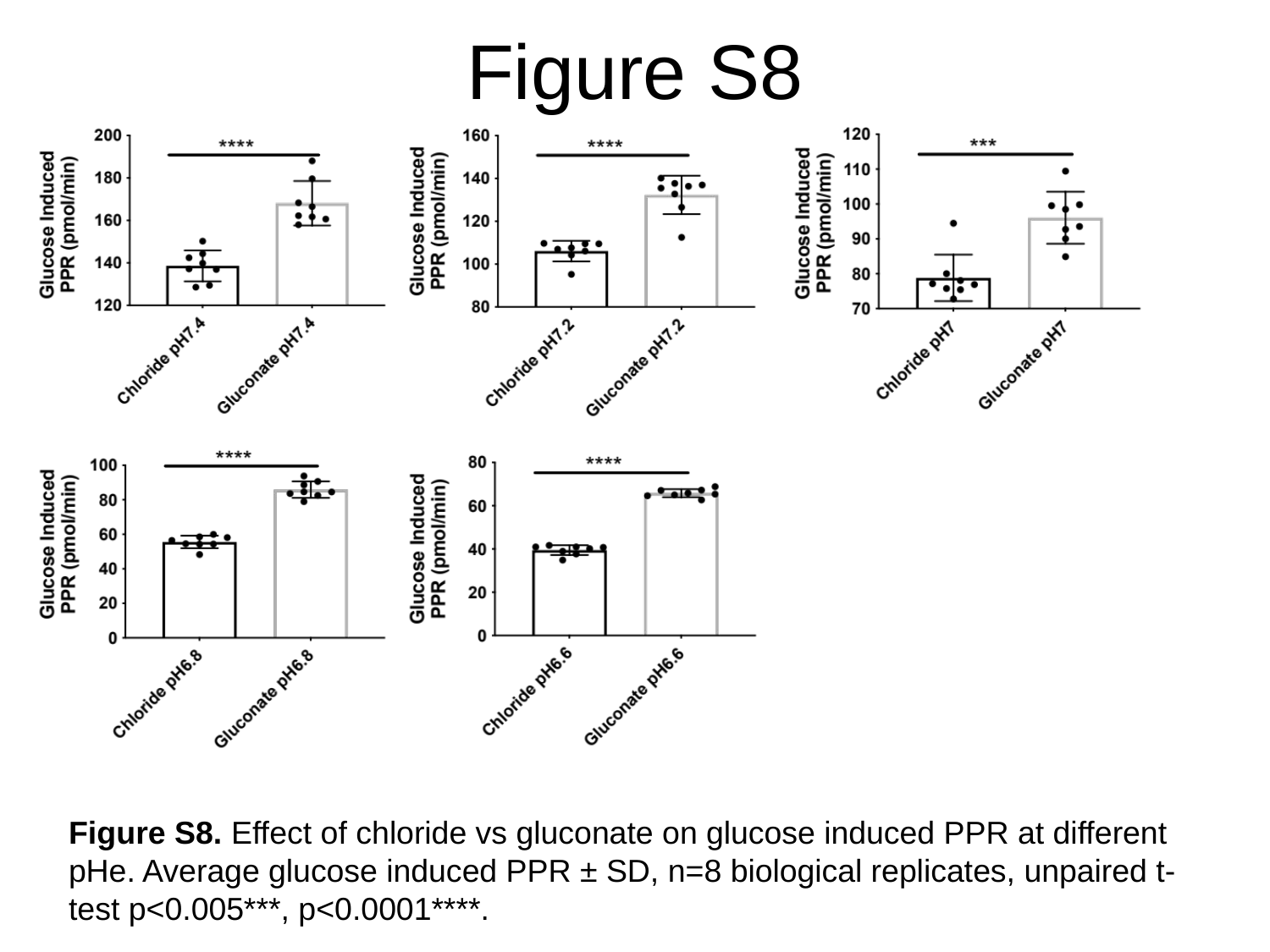

Figure S8
# Figure S8. Effect of chloride vs gluconate on glucose induced PPR at different pHe. Average glucose induced PPR ± SD, n=8 biological replicates, unpaired t-test p<0.005***, p<0.0001****.

## Slide 12
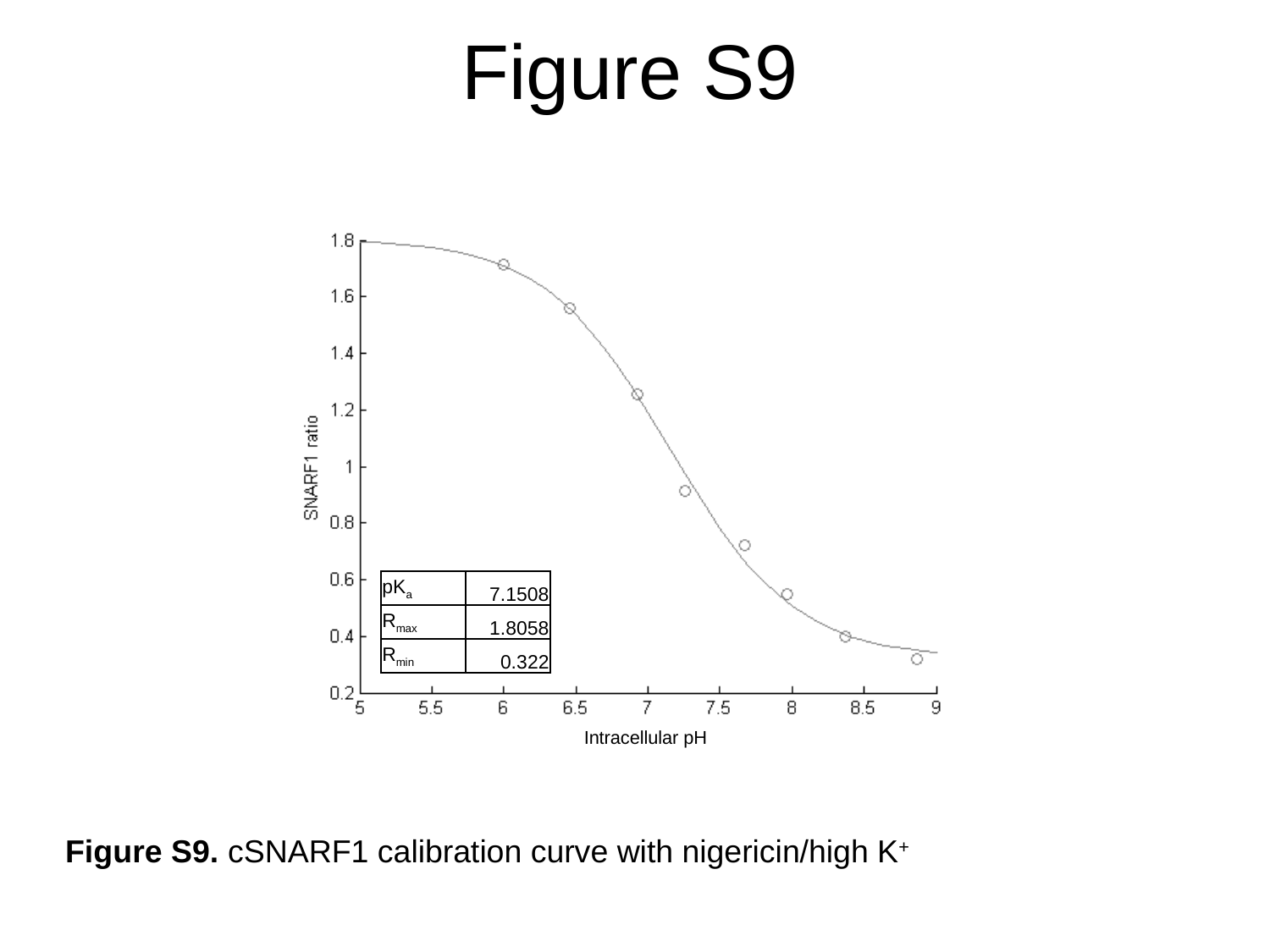

Figure S9
| pKa | 7.1508 |
| --- | --- |
| Rmax | 1.8058 |
| Rmin | 0.322 |
Intracellular pH
Figure S9. cSNARF1 calibration curve with nigericin/high K+

## Slide 13
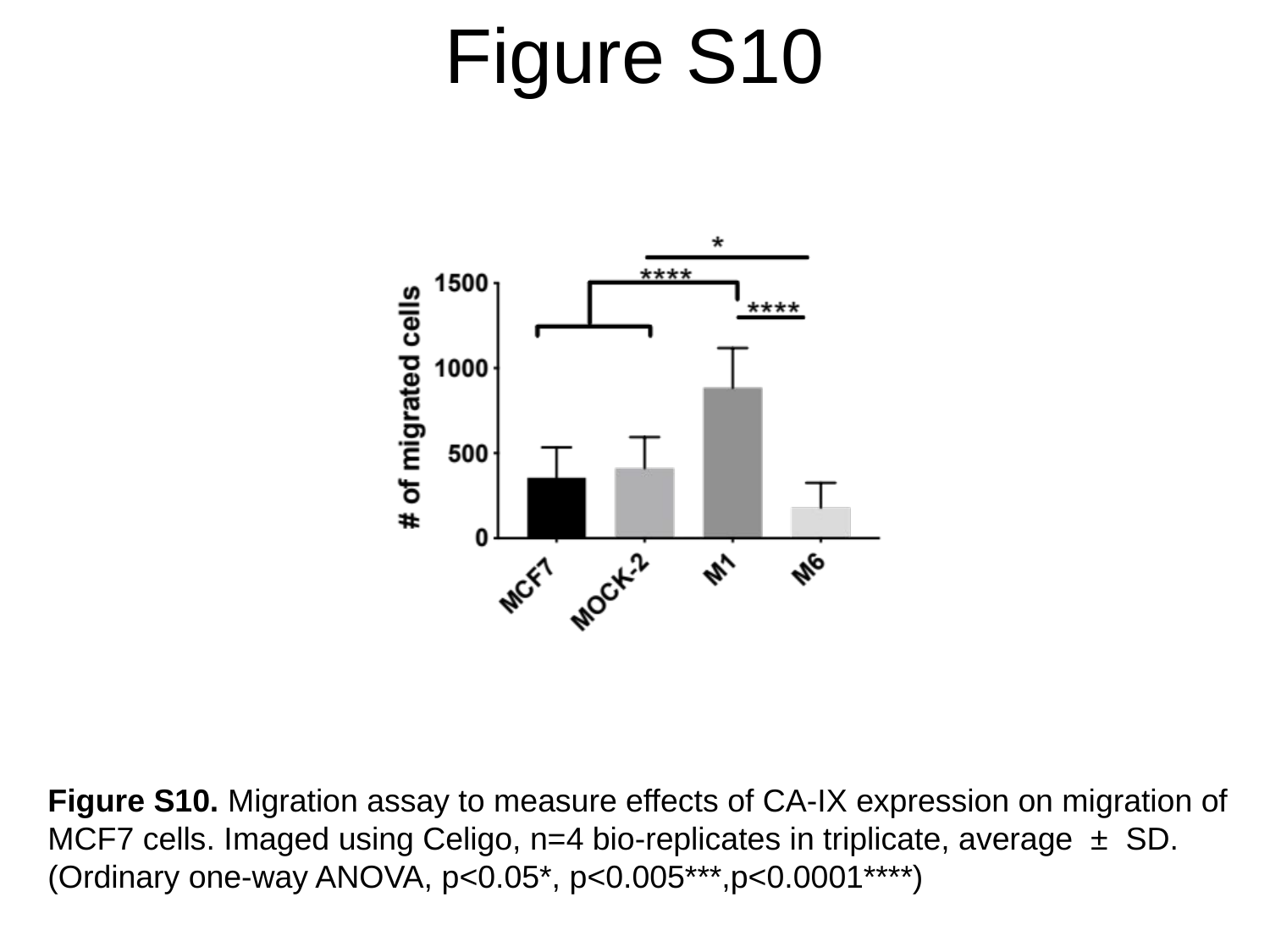

Figure S10
Figure S10. Migration assay to measure effects of CA-IX expression on migration of MCF7 cells. Imaged using Celigo, n=4 bio-replicates in triplicate, average ± SD. (Ordinary one-way ANOVA, p<0.05*, p<0.005***,p<0.0001****)

## Slide 14
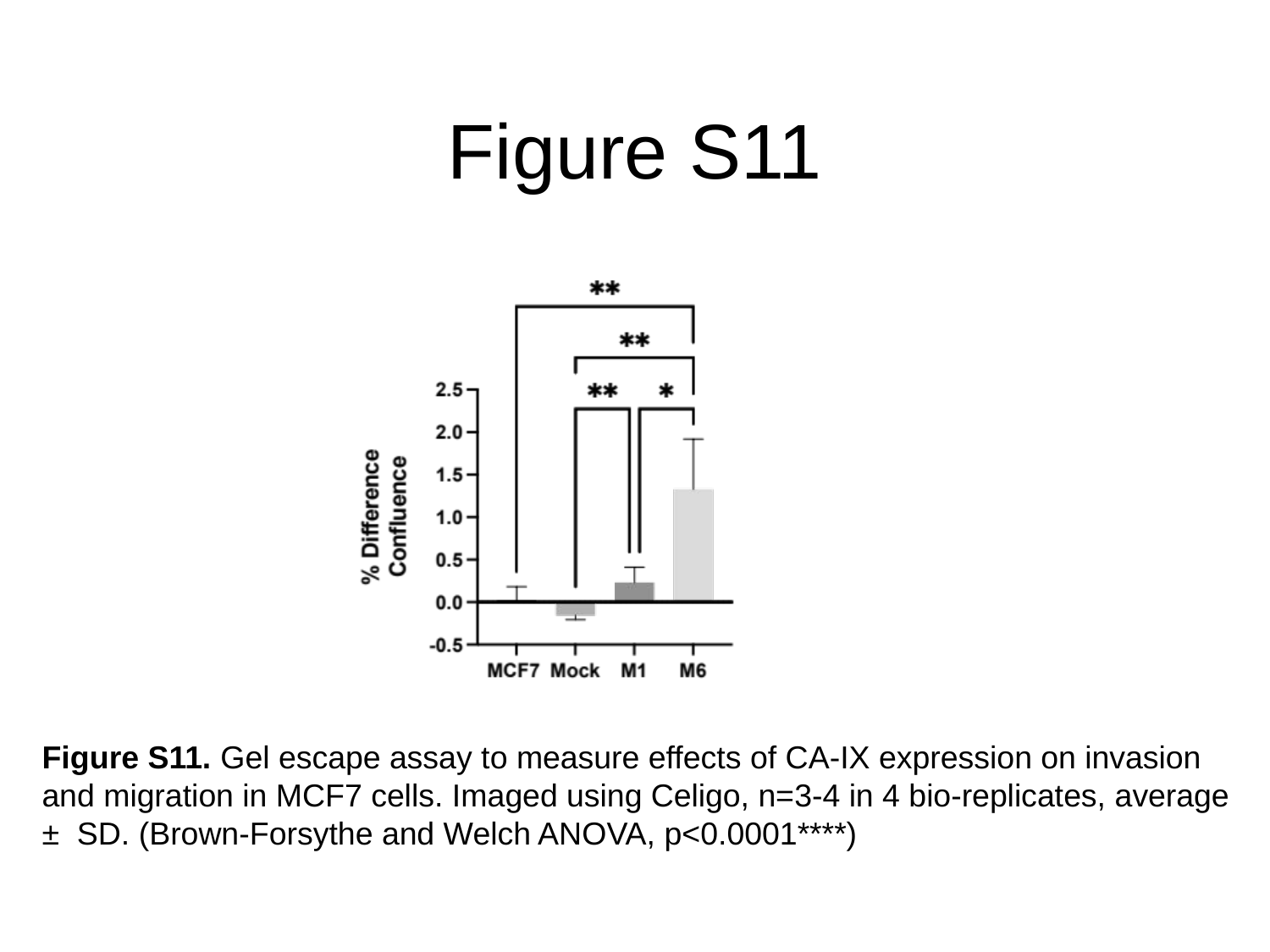

Figure S11
Figure S11. Gel escape assay to measure effects of CA-IX expression on invasion and migration in MCF7 cells. Imaged using Celigo, n=3-4 in 4 bio-replicates, average ± SD. (Brown-Forsythe and Welch ANOVA, p<0.0001****)

## Slide 15
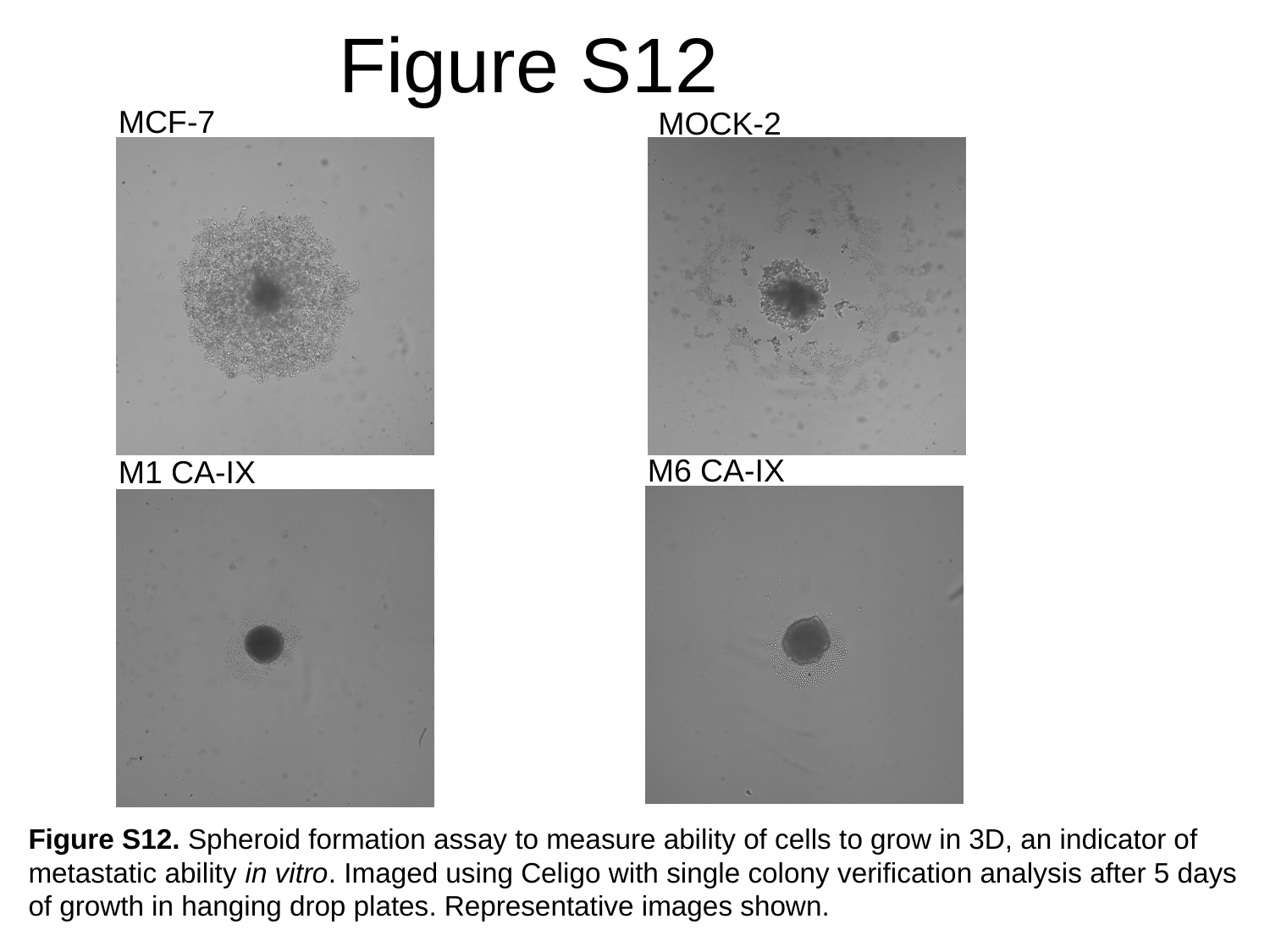

Figure S12
MCF-7
MOCK-2
M6 CA-IX
M1 CA-IX
Figure S12. Spheroid formation assay to measure ability of cells to grow in 3D, an indicator of metastatic ability in vitro. Imaged using Celigo with single colony verification analysis after 5 days of growth in hanging drop plates. Representative images shown.

## Slide 16
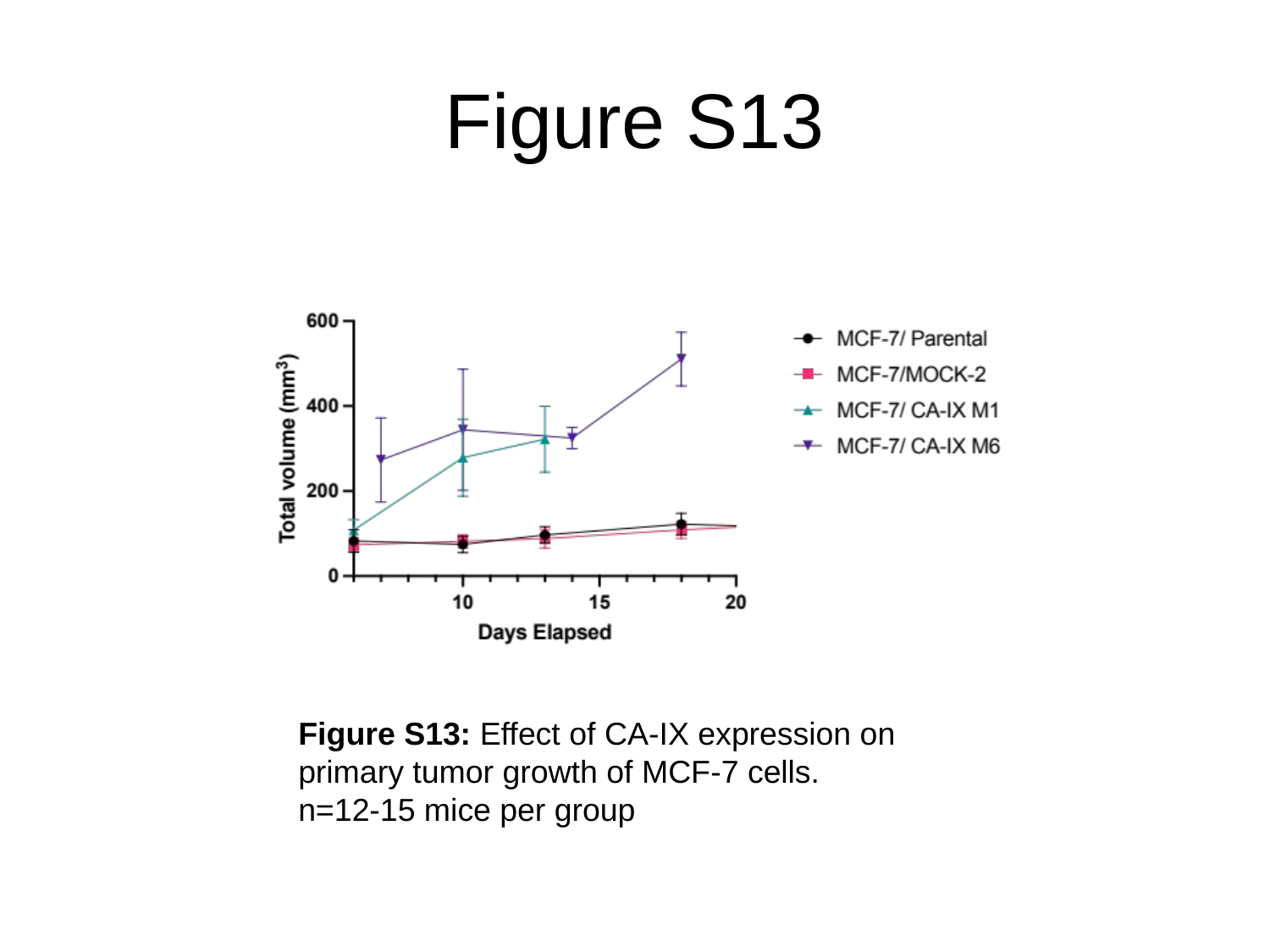

# Figure S13
Figure S13: Effect of CA-IX expression on primary tumor growth of MCF-7 cells. n=12-15 mice per group

## Slide 17
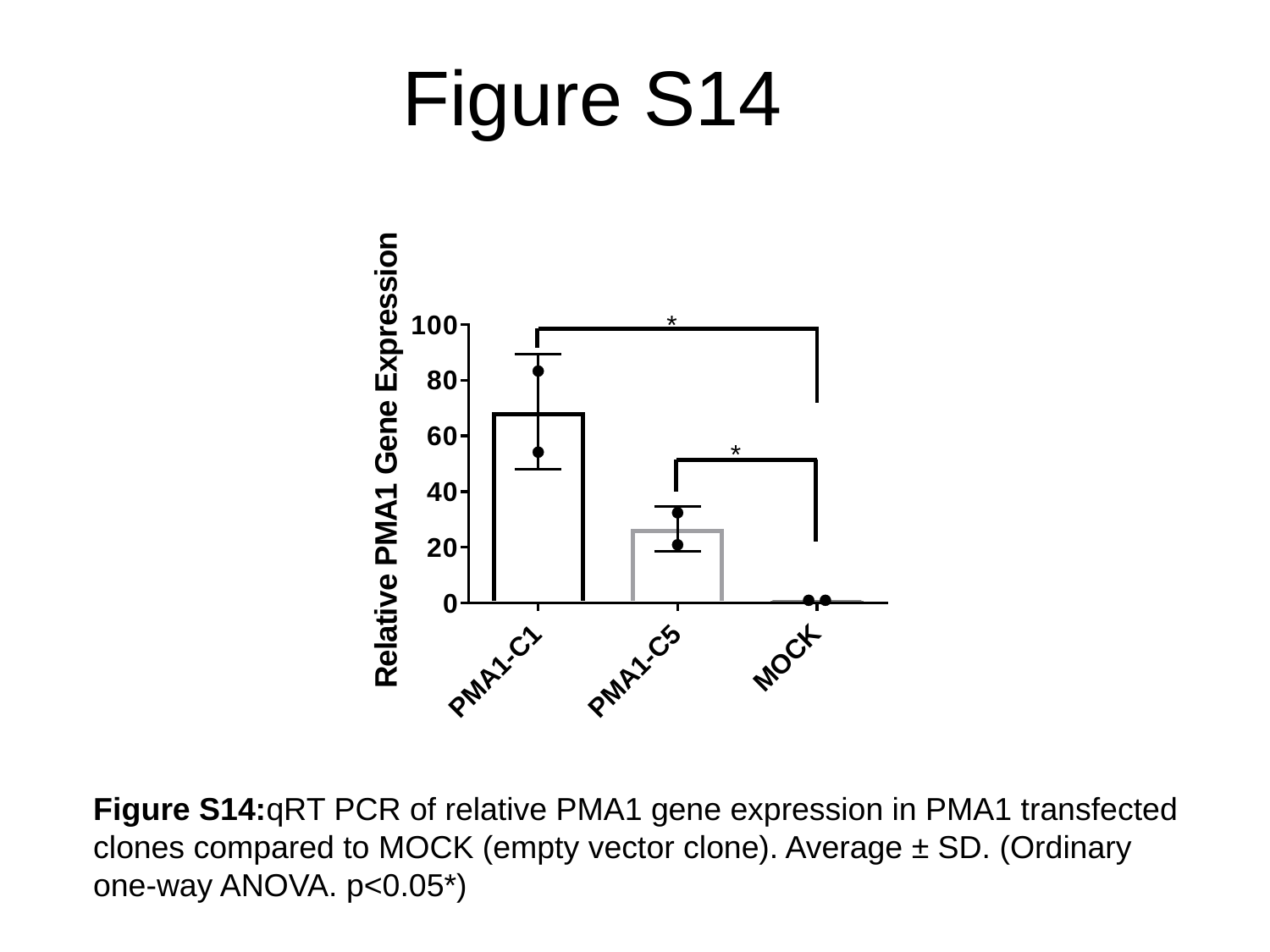

# Figure S14
Figure S14:qRT PCR of relative PMA1 gene expression in PMA1 transfected clones compared to MOCK (empty vector clone). Average ± SD. (Ordinary one-way ANOVA. p<0.05*)

## Slide 18
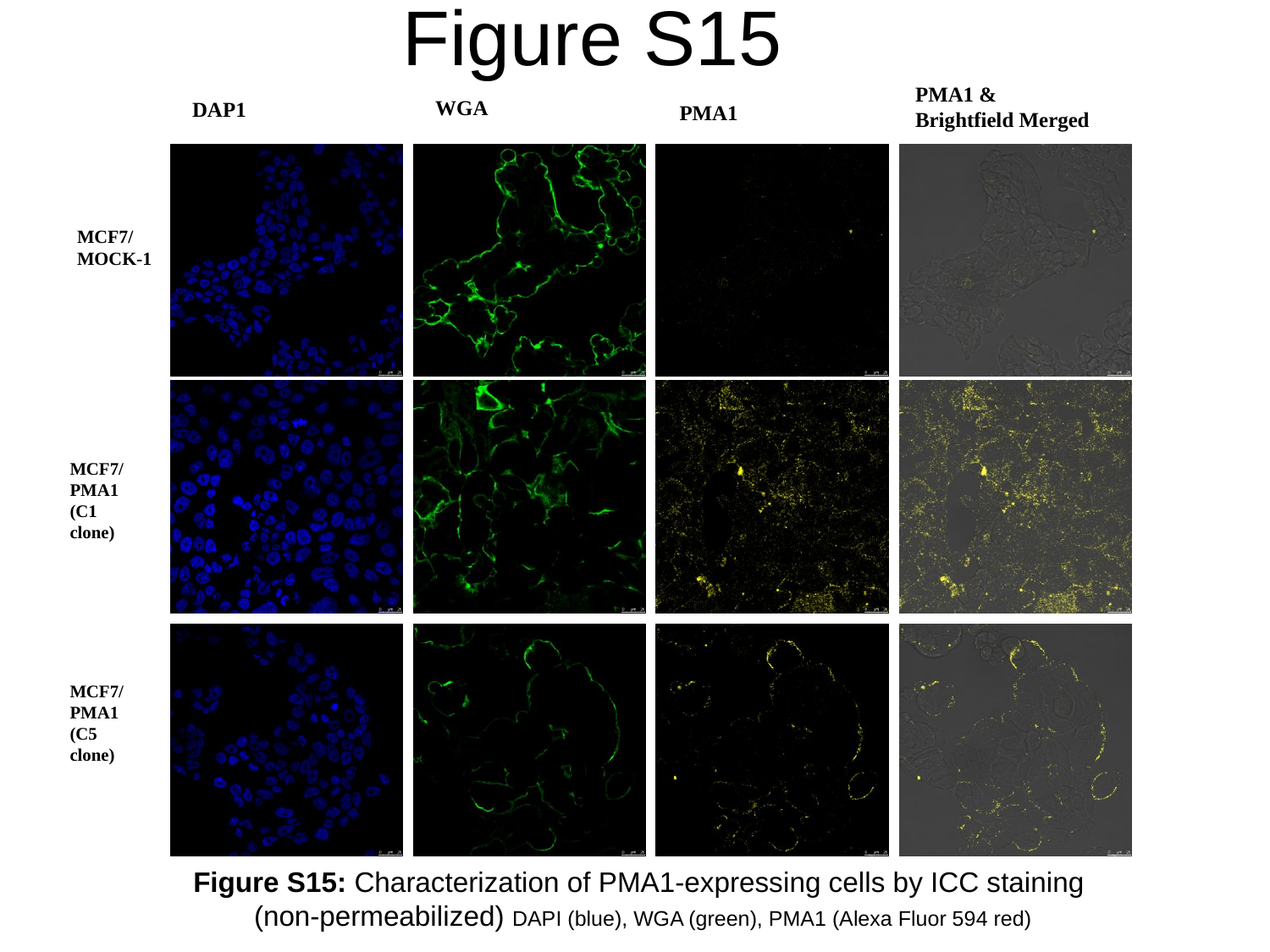

# Figure S15
PMA1 &
Brightfield Merged
WGA
DAP1
PMA1
MCF7/
MOCK-1
MCF7/
PMA1
(C1
clone)
MCF7/
PMA1
(C5
clone)
Figure S15: Characterization of PMA1-expressing cells by ICC staining
(non-permeabilized) DAPI (blue), WGA (green), PMA1 (Alexa Fluor 594 red)

## Slide 19
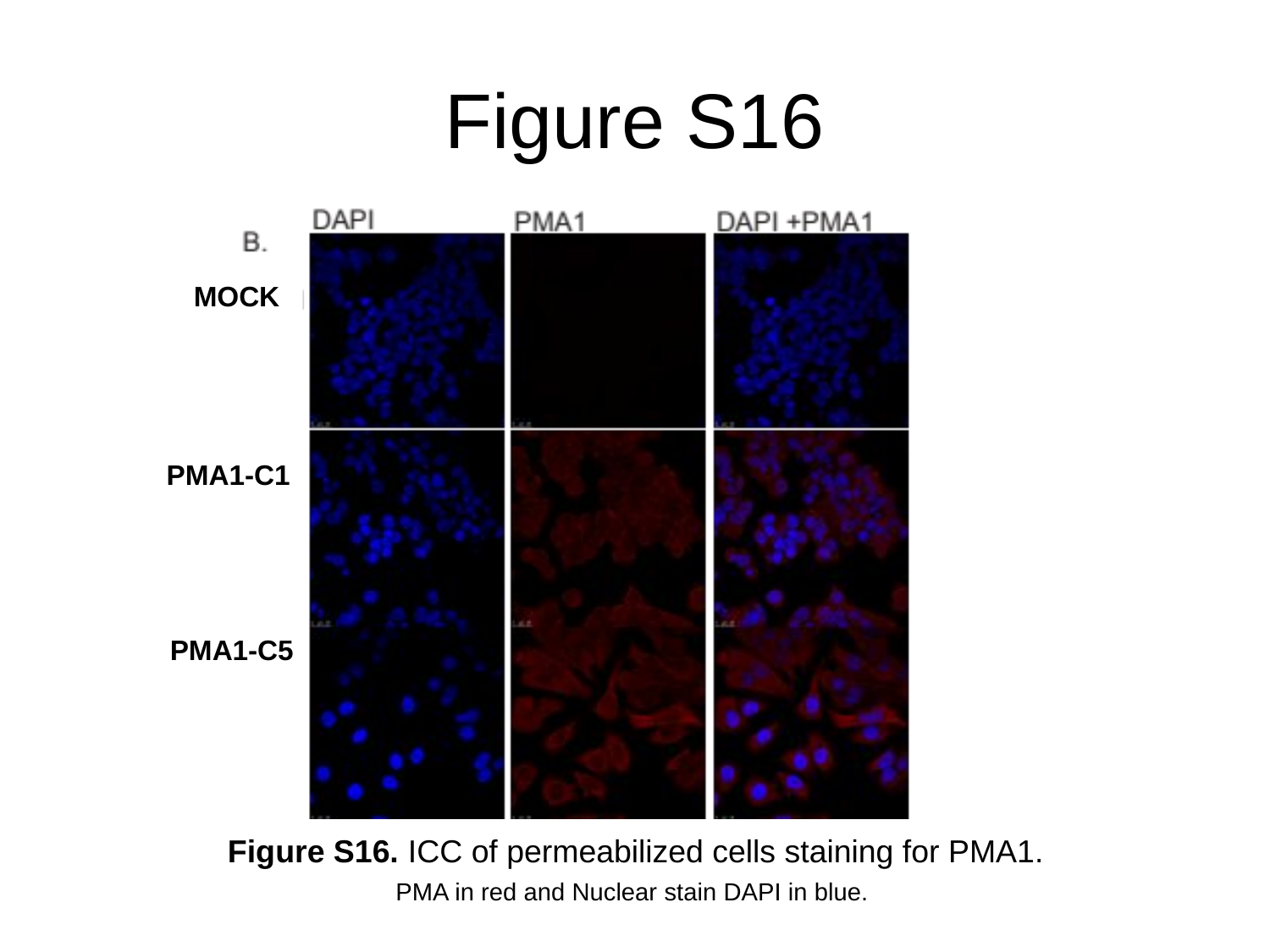

# Figure S16
MOCK
PMA1-C1
PMA1-C5
Figure S16. ICC of permeabilized cells staining for PMA1.
PMA in red and Nuclear stain DAPI in blue.

## Slide 20
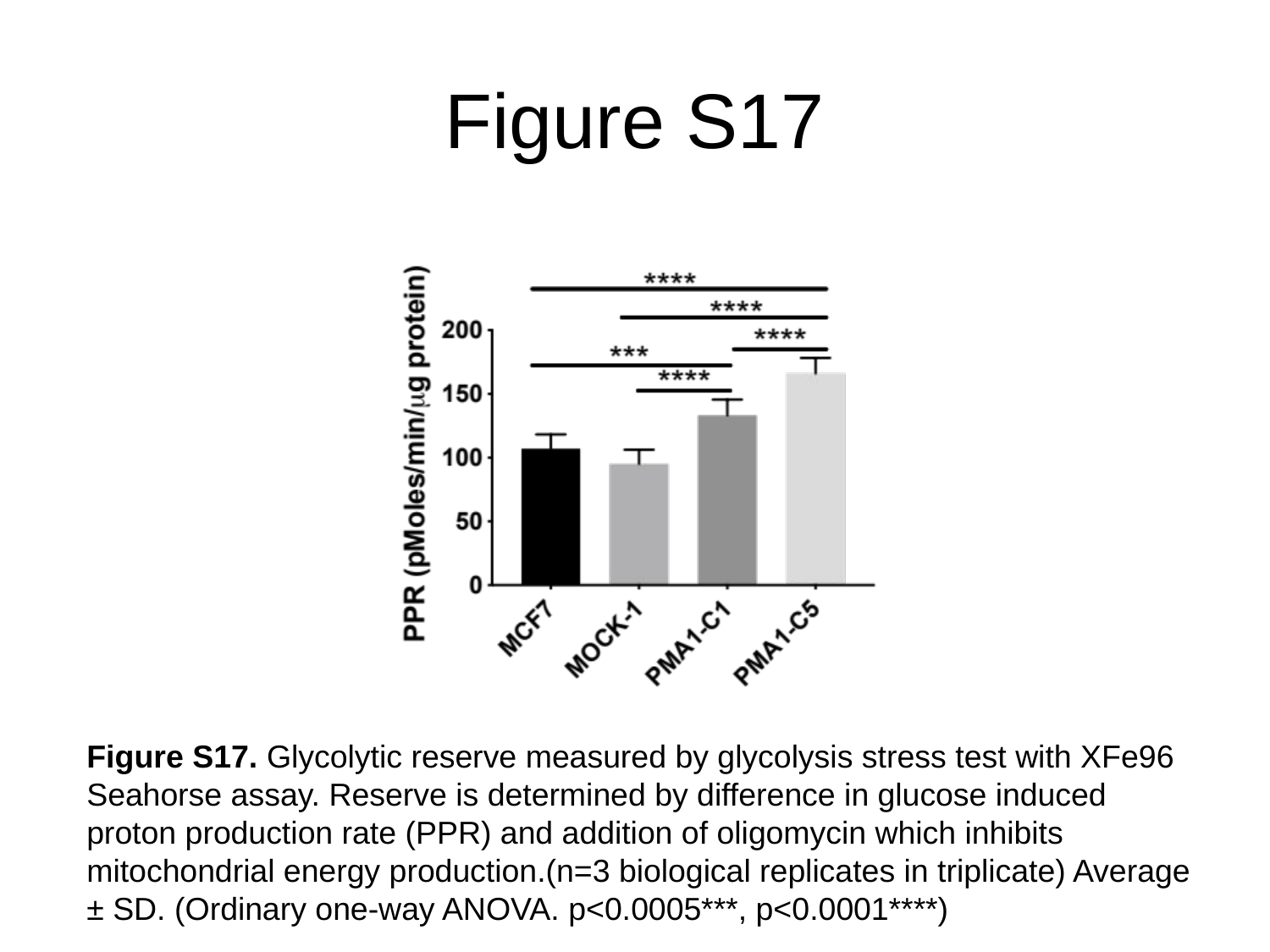

# Figure S17
Figure S17. Glycolytic reserve measured by glycolysis stress test with XFe96 Seahorse assay. Reserve is determined by difference in glucose induced proton production rate (PPR) and addition of oligomycin which inhibits mitochondrial energy production.(n=3 biological replicates in triplicate) Average ± SD. (Ordinary one-way ANOVA. p<0.0005***, p<0.0001****)

## Slide 21
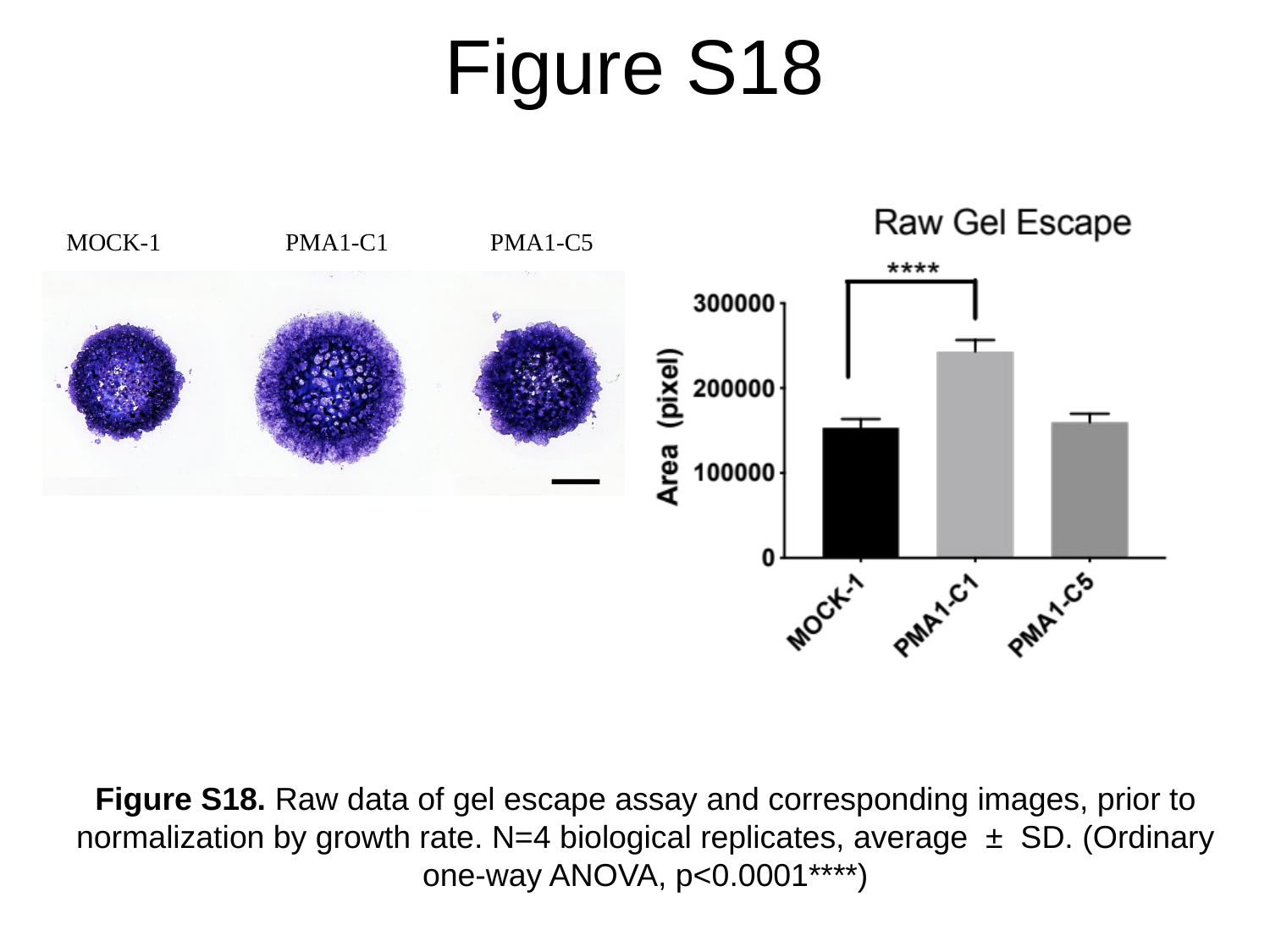

# Figure S18
MOCK-1	 PMA1-C1	 PMA1-C5
Figure S18. Raw data of gel escape assay and corresponding images, prior to normalization by growth rate. N=4 biological replicates, average ± SD. (Ordinary one-way ANOVA, p<0.0001****)

## Slide 22
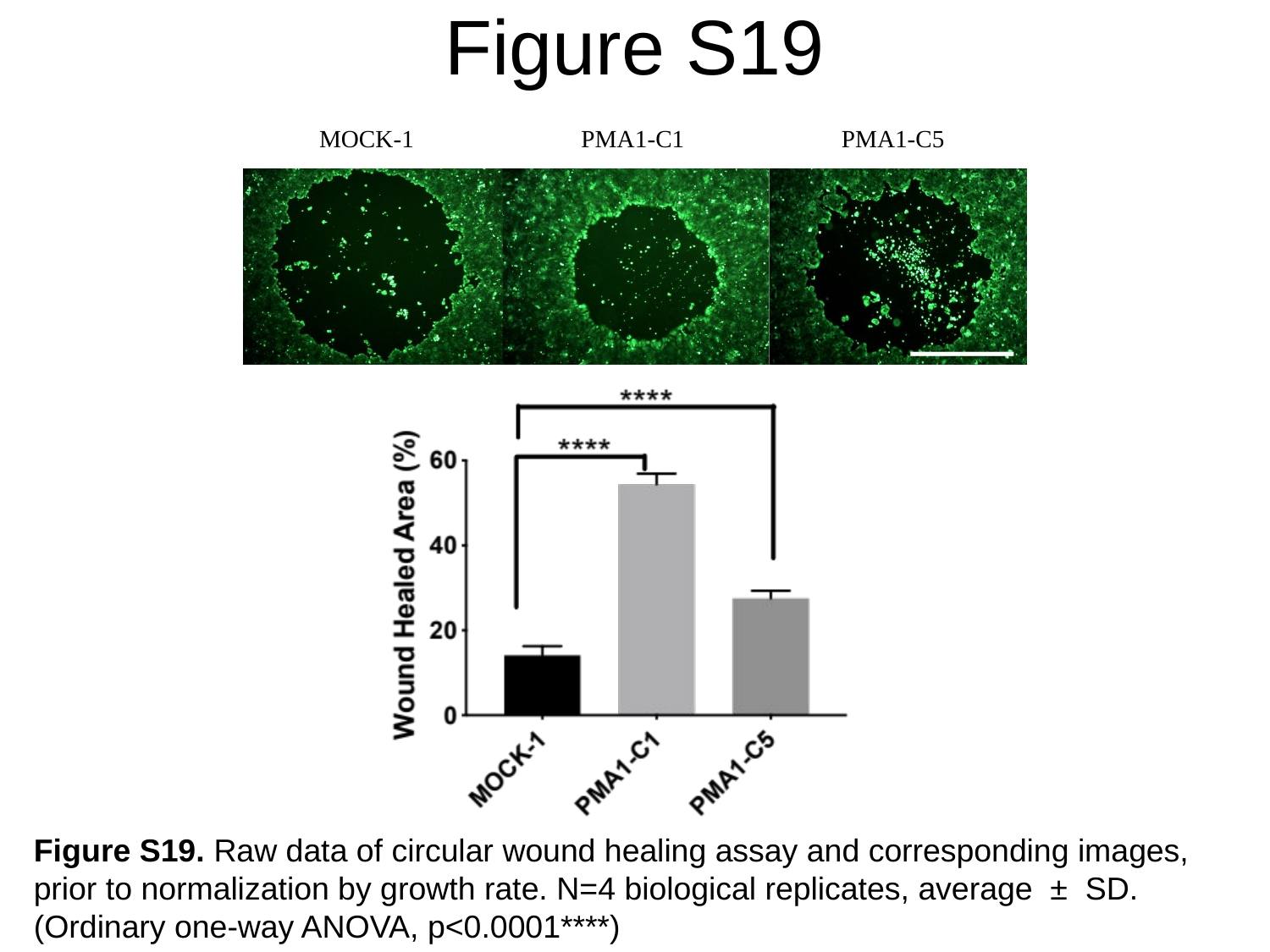

# Figure S19
MOCK-1	 PMA1-C1	 PMA1-C5
Figure S19. Raw data of circular wound healing assay and corresponding images, prior to normalization by growth rate. N=4 biological replicates, average ± SD. (Ordinary one-way ANOVA, p<0.0001****)

## Slide 23
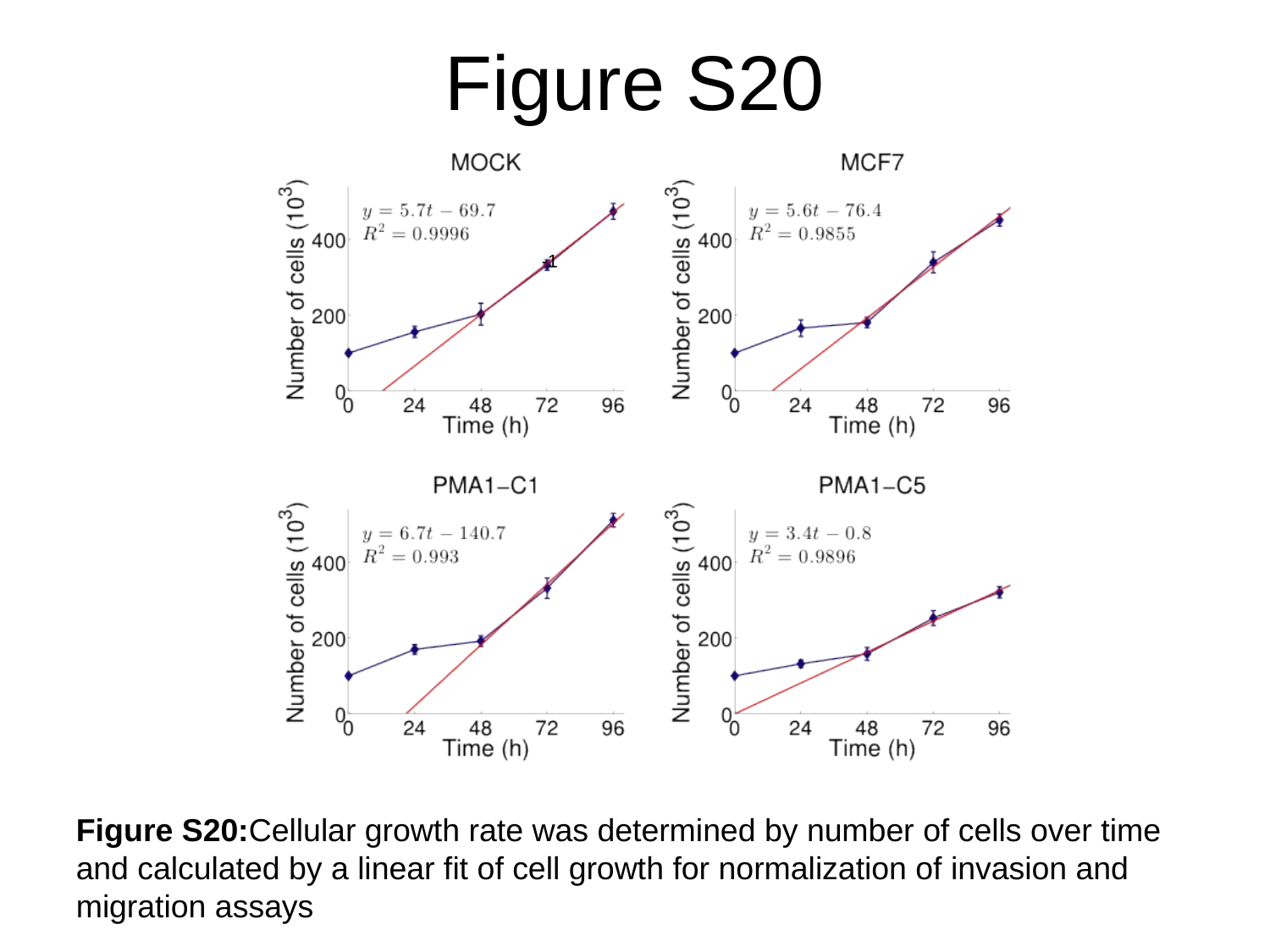

# Figure S20
-1
Figure S20:Cellular growth rate was determined by number of cells over time and calculated by a linear fit of cell growth for normalization of invasion and migration assays

## Slide 24
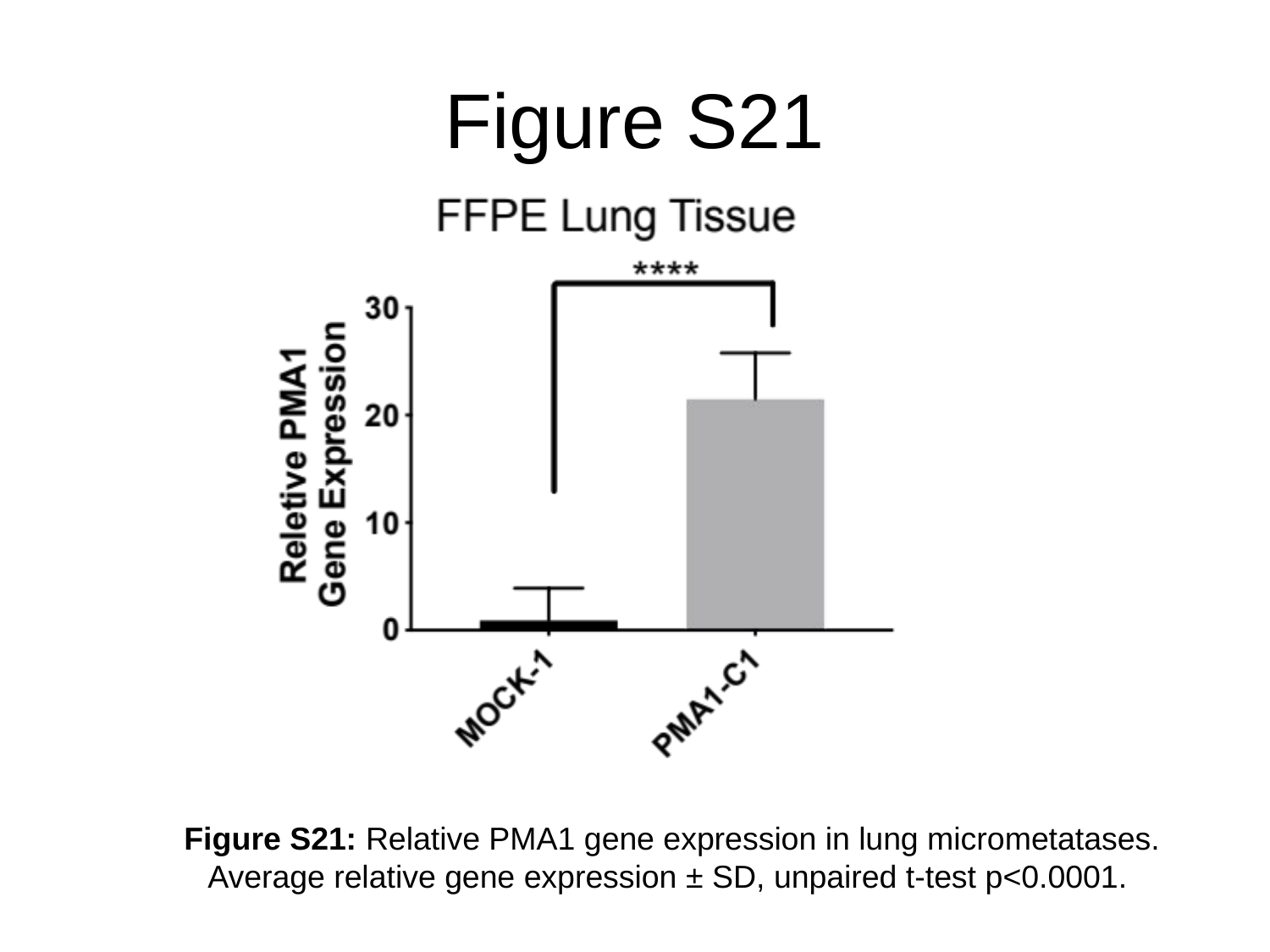

# Figure S21
Figure S21: Relative PMA1 gene expression in lung micrometatases. Average relative gene expression ± SD, unpaired t-test p<0.0001.

## Slide 25
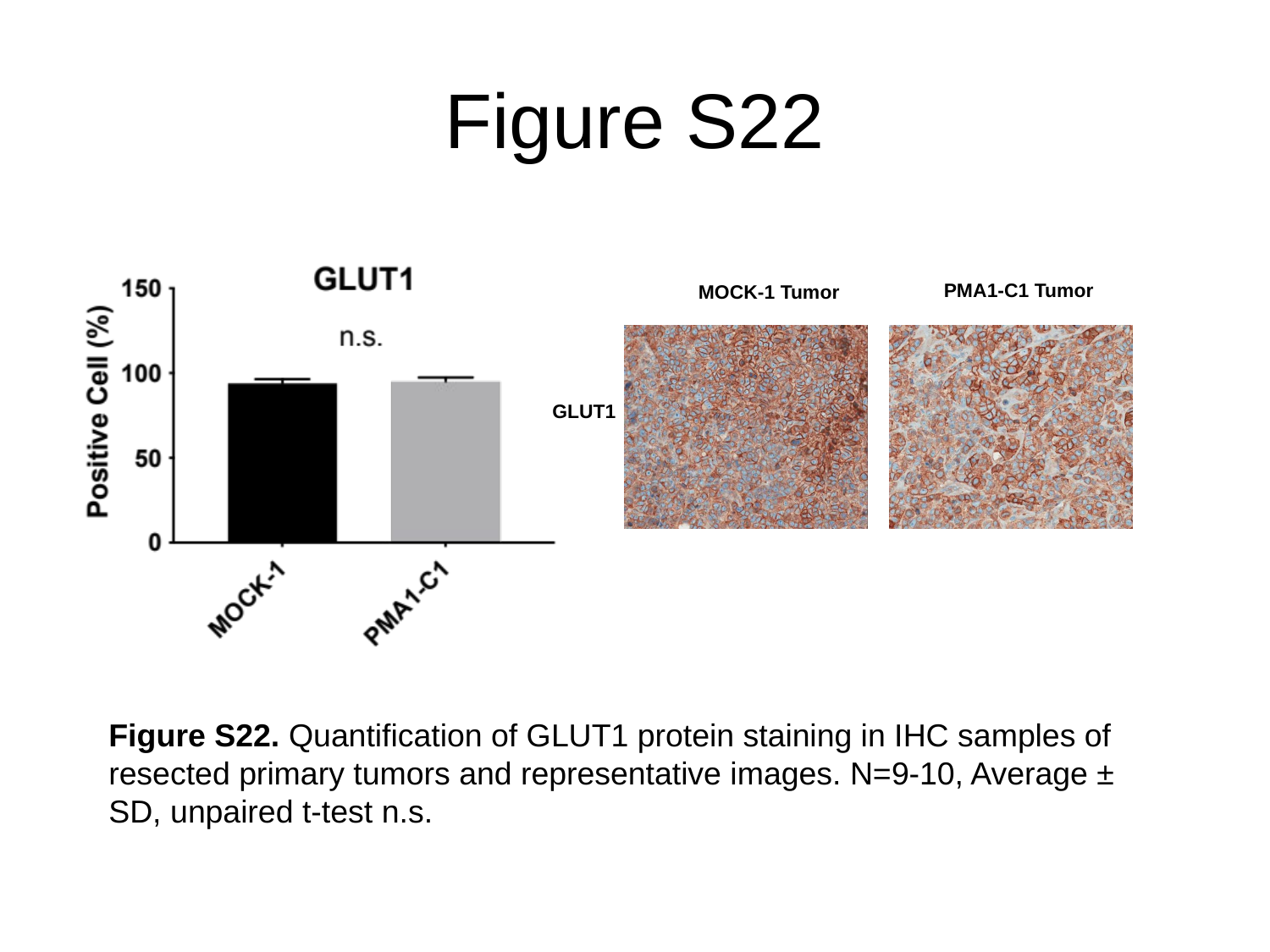

# Figure S22
PMA1-C1 Tumor
MOCK-1 Tumor
GLUT1
Figure S22. Quantification of GLUT1 protein staining in IHC samples of resected primary tumors and representative images. N=9-10, Average ± SD, unpaired t-test n.s.

## Slide 26
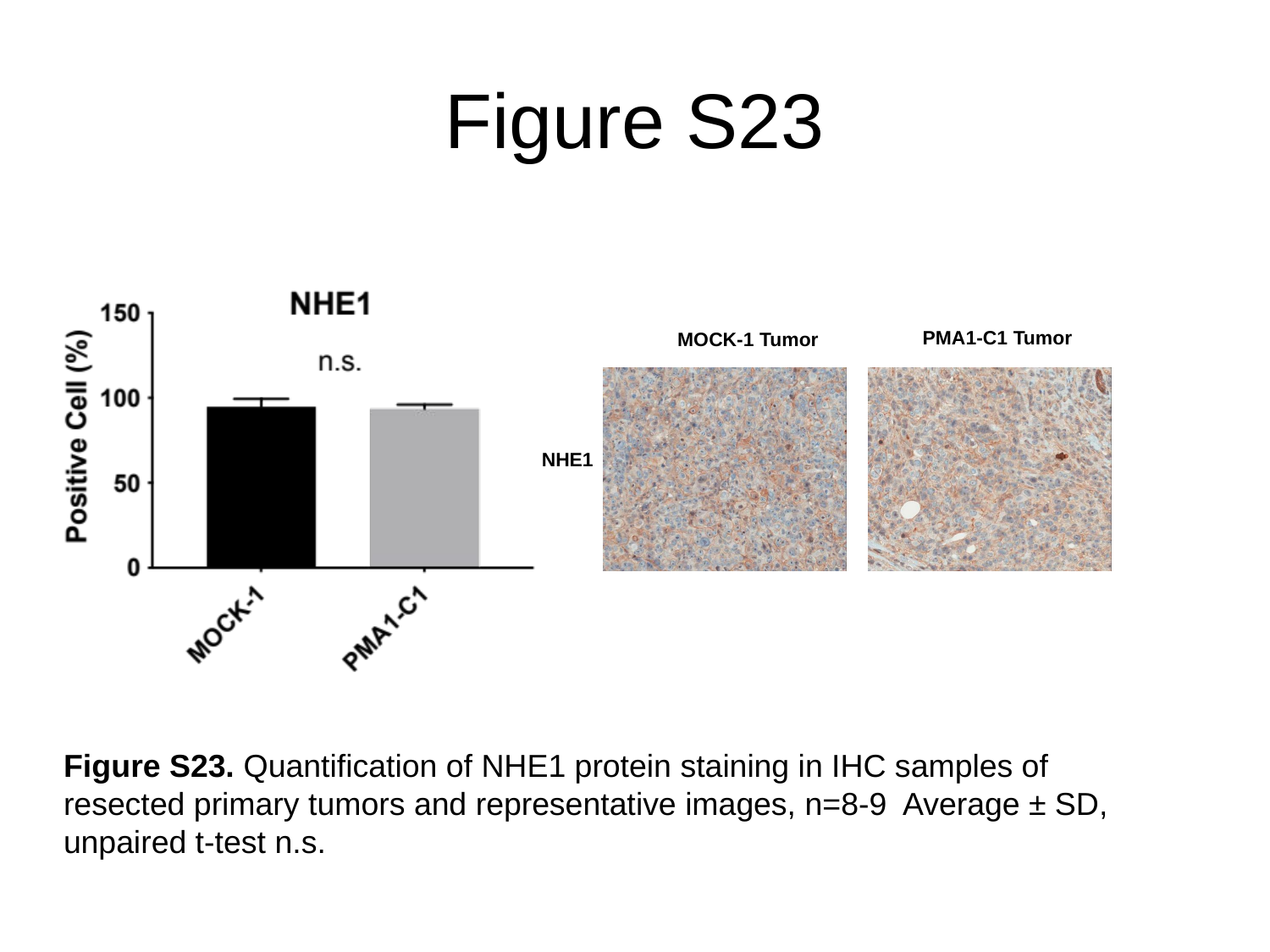

# Figure S23
PMA1-C1 Tumor
MOCK-1 Tumor
NHE1
Figure S23. Quantification of NHE1 protein staining in IHC samples of resected primary tumors and representative images, n=8-9 Average ± SD, unpaired t-test n.s.

## Slide 27
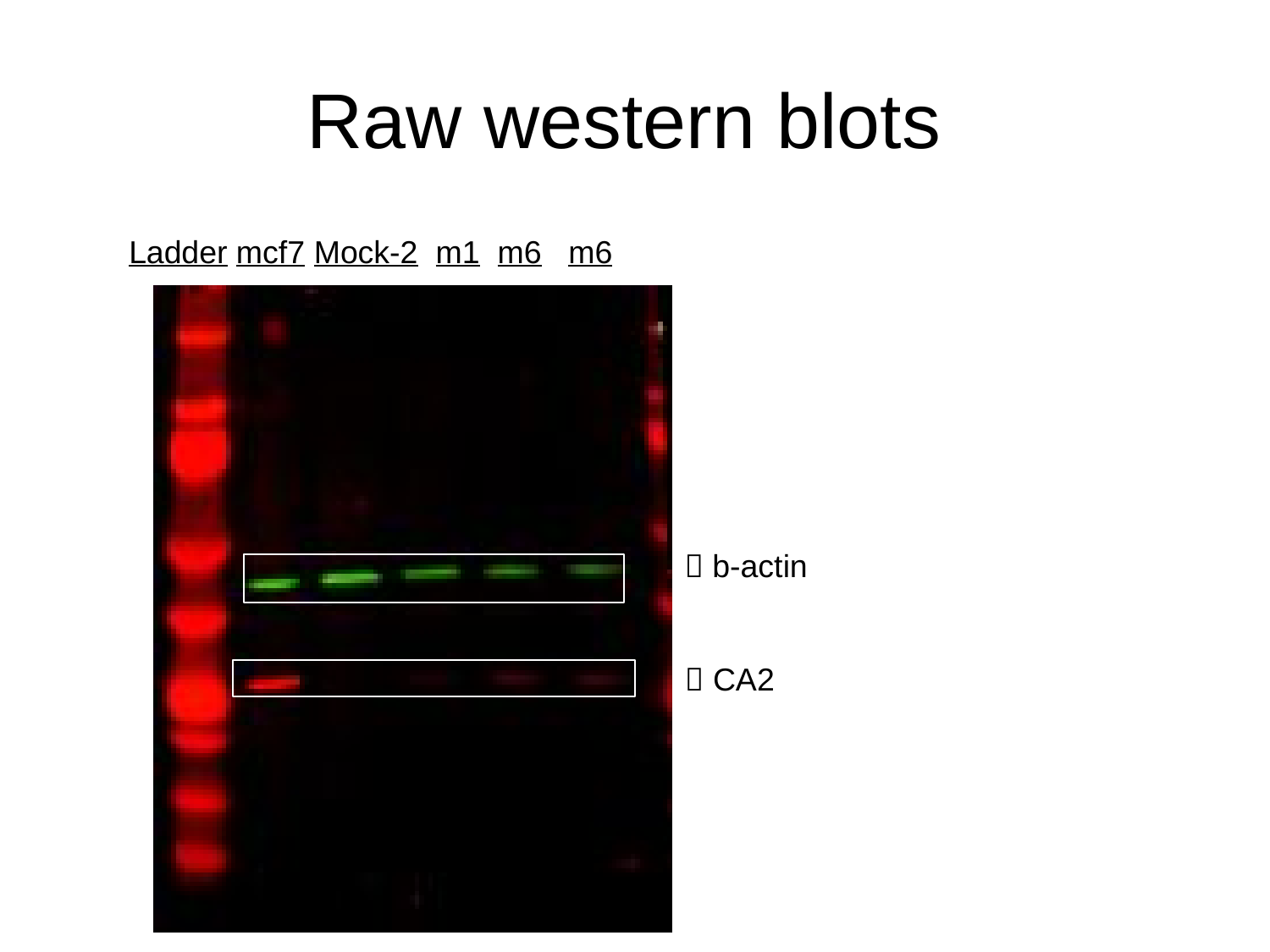

# Raw western blots
Ladder mcf7 Mock-2 m1 m6 m6
 b-actin
 CA2

## Slide 28
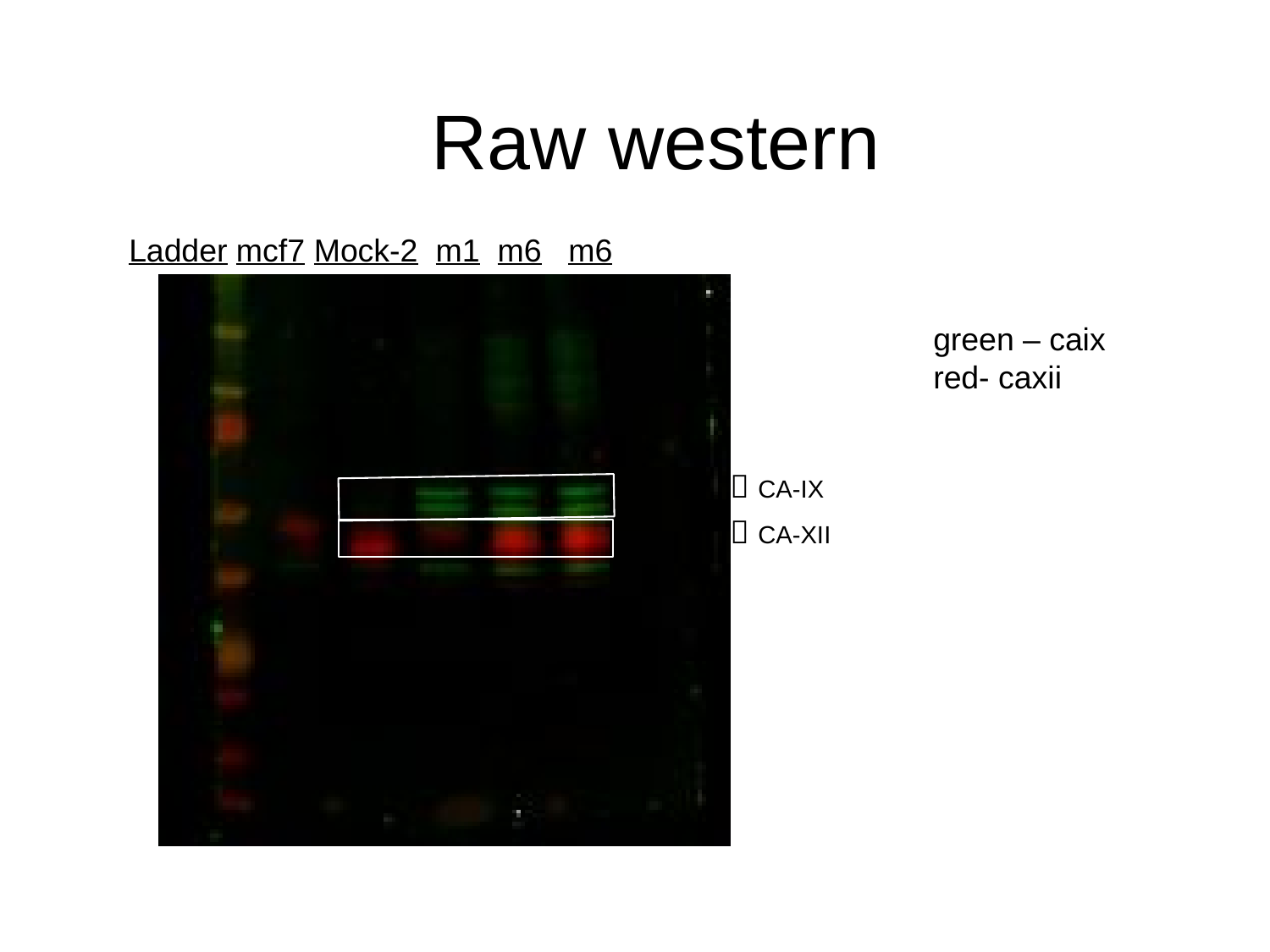

Raw western
Ladder mcf7 Mock-2 m1 m6 m6
green – caix
red- caxii
 CA-IX
 CA-XII

## Slide 29
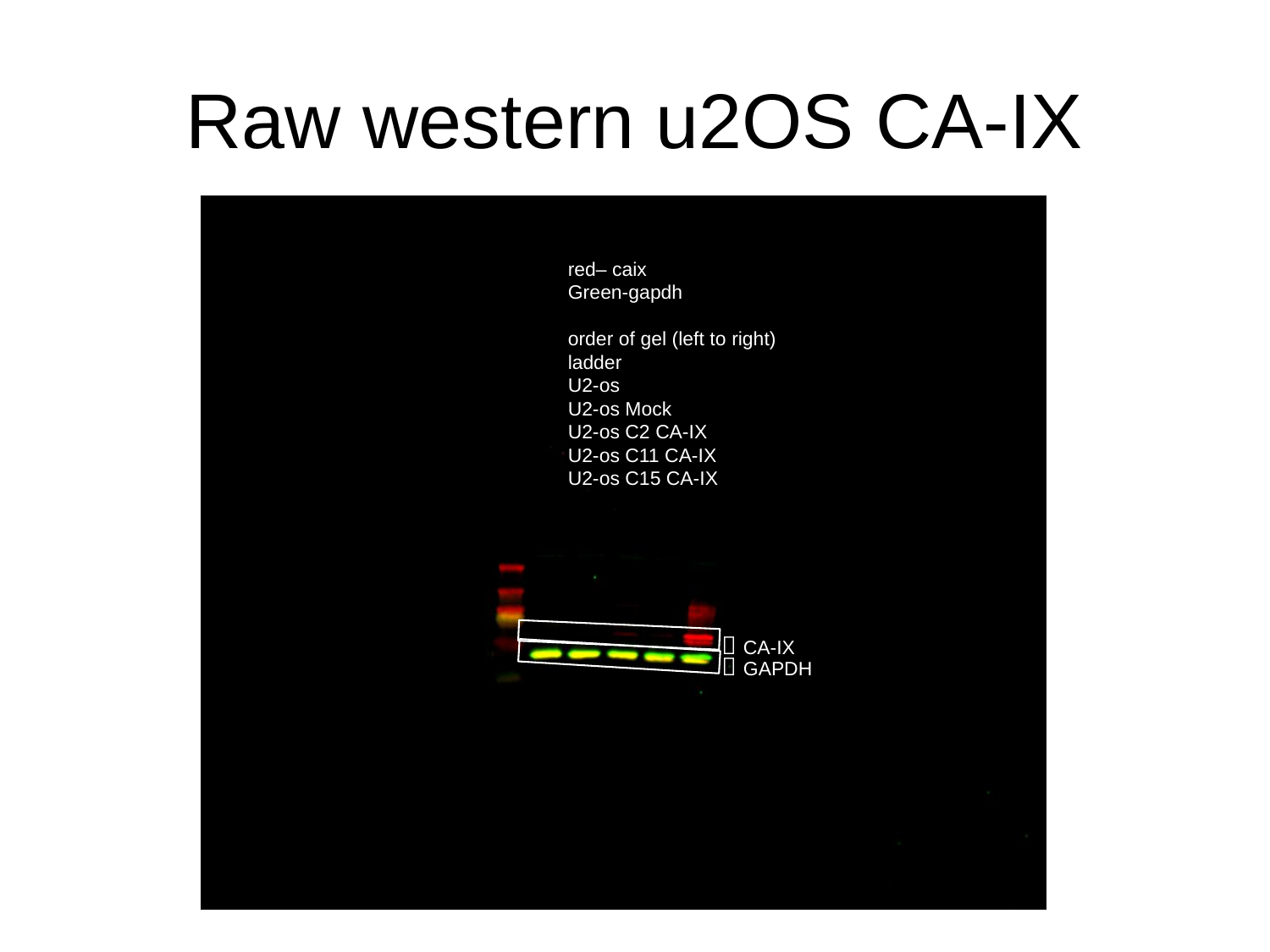

# Raw western u2OS CA-IX
red– caix
Green-gapdh
order of gel (left to right)
ladder
U2-os
U2-os Mock
U2-os C2 CA-IX
U2-os C11 CA-IX
U2-os C15 CA-IX
 CA-IX
 GAPDH

## Slide 30
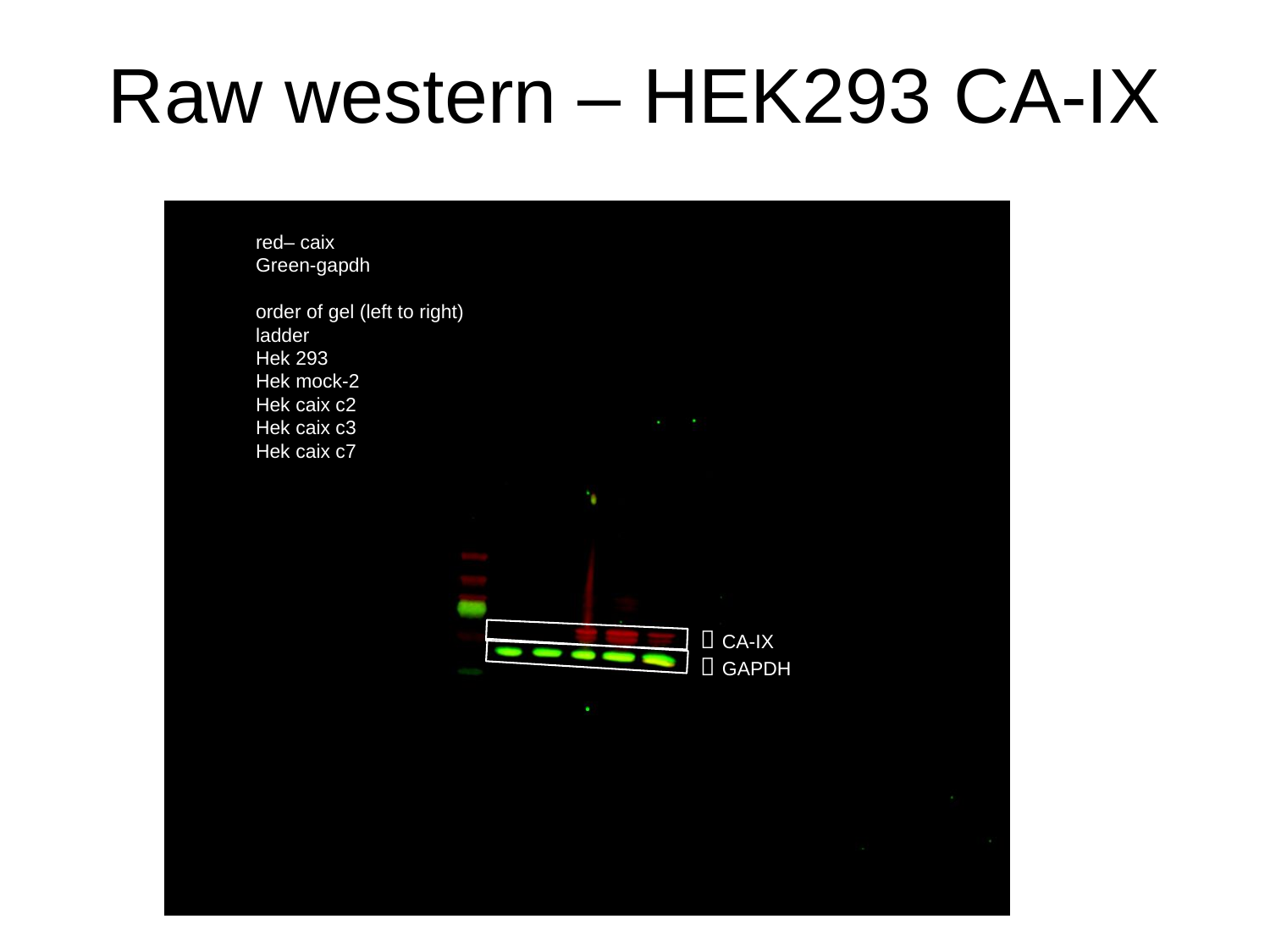

Raw western – HEK293 CA-IX
red– caix
Green-gapdh
order of gel (left to right)
ladder
Hek 293
Hek mock-2
Hek caix c2
Hek caix c3
Hek caix c7
 CA-IX
 GAPDH

## Slide 31
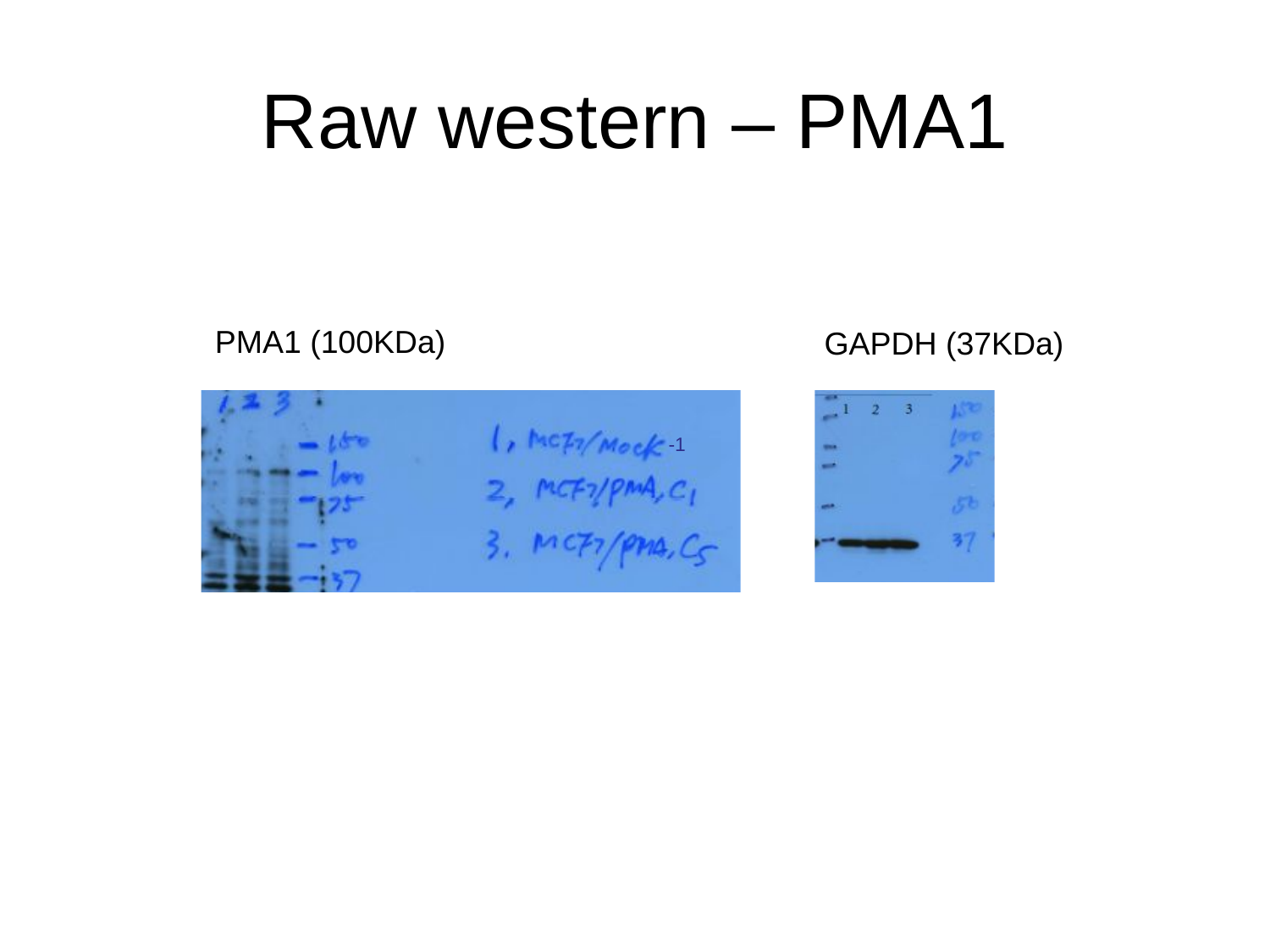

# Raw western – PMA1
PMA1 (100KDa)
GAPDH (37KDa)
-1

## Slide 32
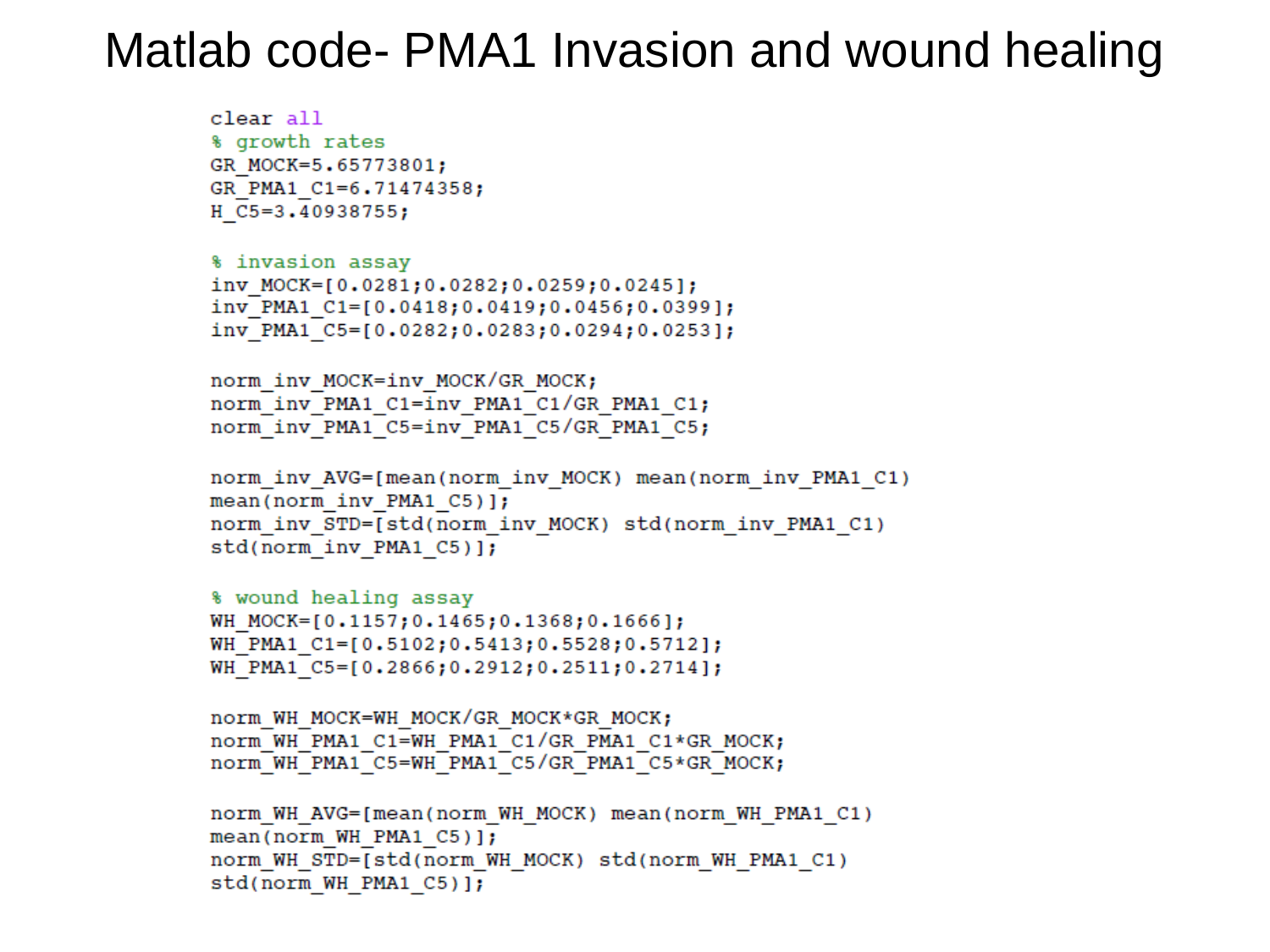

# Matlab code- PMA1 Invasion and wound healing

## Slide 33
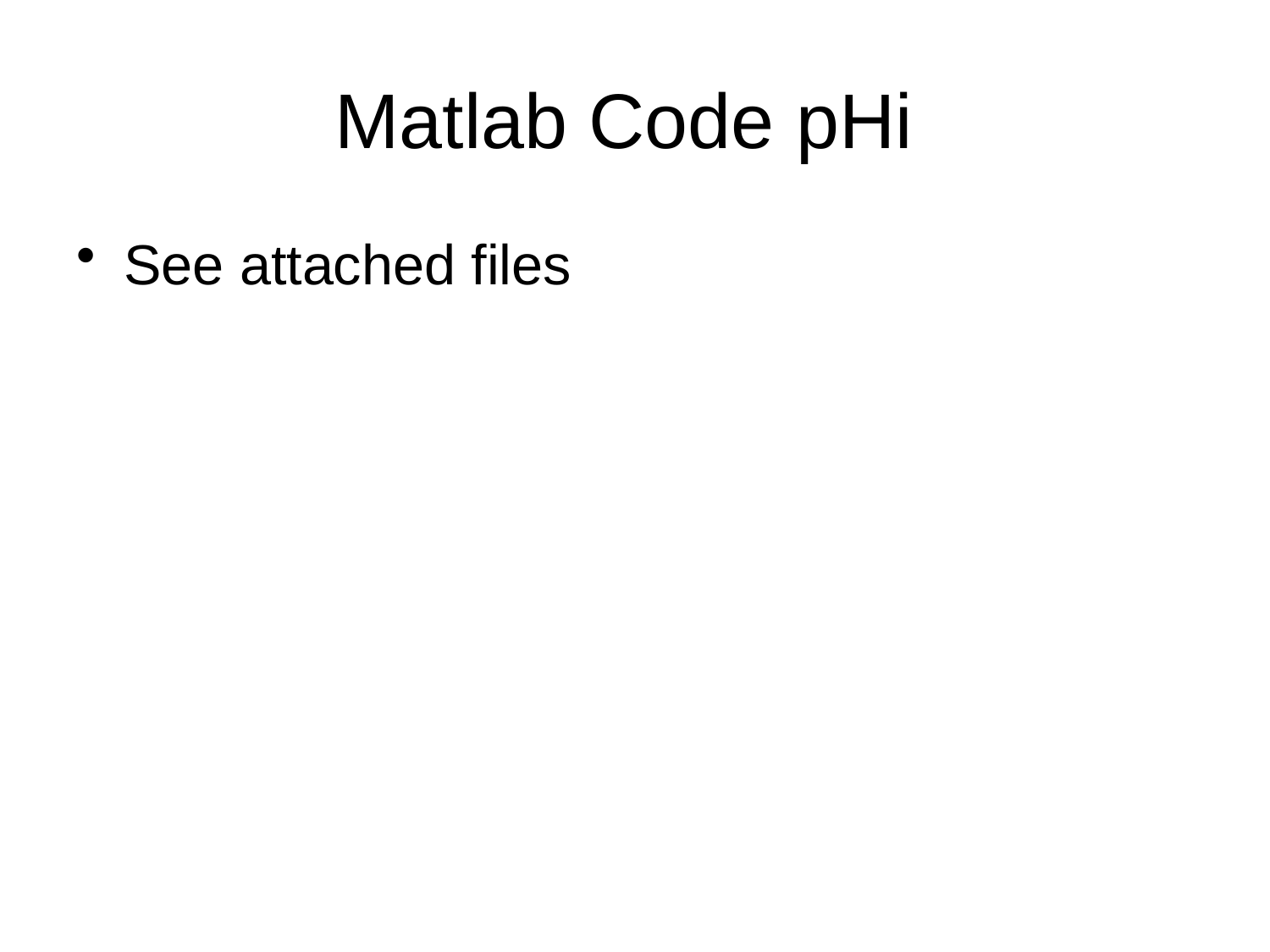

# Matlab Code pHi
See attached files
